# Crystal structure of a homotrimeric verrucomicrobial *exo*-*β*-1,4-mannosidase active in the hindgut of the wood-feeding termite *Reticulitermes flavipes*

**DOI:** 10.1016/j.yjsbx.2021.100048

**Published:** 2021-05-24

**Authors:** Dayanand C. Kalyani, Tom Reichenbach, Markus M. Keskitalo, Julian Conrad, Henrik Aspeborg, Christina Divne

**Affiliations:** a School of Engineering Sciences in Chemistry, Biotechnology, and Health (CBH), KTH Royal Institute of Technology, Roslagstullsbacken 21, SE-11421 Stockholm, Sweden; b Department of Biochemistry and Biophysics, Stockholm University, Svante Arrhenius väg 16C, SE-10691 Stockholm, Sweden; c Science for Life Laboratory, Stockholm University, Tomtebodavägen 23, SE-17165 Solna, Sweden

**Keywords:** CAZy, Carbohydrate-Active enZymes database, CMC, carboxymethyl cellulose, cryo-EM, electron cryo-microscopy, DP, degree of polymerization, EDTA, ethylenediaminetetraacetic acid, ESI-MS, electrospray ionization mass spectrometry, Fuc, fucopyranoside, Gal, galactopyranoside, GH, glycoside hydrolase, Glc, glucopyranoside, GlcNAc, N-acetyl glucosamine, HEPES, 4-(2-hydroxyethyl)-1-piperazineethanesulfonic acid, HPAEC-PAD, High Performance Anion Exchange Chromatography and Pulsed Amperometric Detection, IPTG, β-D-1-thiogalactopyranoside, LBG, locust bean gum, Man, mannopyranoside, MOS, mannooligosaccharides, MWCO, molecular weight cut-off, Op5Man5, *exo*-*β*-1,4-mannosidase from *Opitutaceae* bacterium strain TAV5, *p*NP, p-nitrophenyl, SDS-PAGE, sodium dodecyl sulfate–polyacrylamide gel electrophoresis, SEC, size-exclusion chromatography, TCEP, tris (2-carboxyethyl) phosphine hydrochloride, TLC, thin-layer chromatography, Xyl, xylopyranoside, Exo-*β*-1, 4-mannosidase, Glycosyl hydrolase family 5, Termite hindgut, Crystal structure, Electron cryo-microscopy, *Reticulitermes flavipes*, Verrucomicrobia, *Opitutaceae*

## Abstract

The termite *Reticulitermes flavipes* causes extensive damage due to the high efficiency and broad specificity of the ligno- and hemicellulolytic enzyme systems produced by its symbionts. Thus, the *R. flavipes* gut microbiome is expected to constitute an excellent source of enzymes that can be used for the degradation and valorization of plant biomass. The symbiont *Opitutaceae* bacterium strain TAV5 belongs to the phylum Verrucomicrobia and thrives in the hindgut of *R. flavipes*. The sequence of the gene with the locus tag opit5_10225 in the *Opitutaceae* bacterium strain TAV5 genome has been classified as a member of glycoside hydrolase family 5 (GH5), and provisionally annotated as an *endo*-*β*-mannanase. We characterized biochemically and structurally the opit5_10225 gene product, and show that the enzyme, *Op5*Man5, is an *exo*-*β*-1,4-mannosidase [EC 3.2.1.25] that is highly specific for *β*-1,4-mannosidic bonds in mannooligosaccharides and ivory nut mannan. The structure of *Op5*Man5 was phased using electron cryo-microscopy and further determined and refined at 2.2 Å resolution using X-ray crystallography. *Op5*Man5 features a 200-kDa large homotrimer composed of three modular monomers. Despite insignificant sequence similarity, the structure of the monomer, and homotrimeric assembly are similar to that of the GH42-family *β*-galactosidases and the GH164-family *exo*-*β*-1,4-mannosidase *Bs*164 from *Bacteroides salyersiae*. To the best of our knowledge *Op5*Man5 is the first structure of a glycoside hydrolase from a bacterial symbiont isolated from the *R. flavipes* digestive tract, as well as the first example of a GH5 glycoside hydrolase with a GH42 *β*-galactosidase-type homotrimeric structure.

## Highlights

• First structure of a glycoside hydrolase from a bacterial symbiont isolated from the digestive tract of the notorious termite pest *Reticulitermes flavipes.*
• First example of a GH5 glycoside hydrolase that features a GH42-type homotrimeric structure.
• High *exo*-type specificity for the terminal ®-1,4-mannosidic linkages in mannooligosaccharides and unsubstituted®-mannans.
• Verrucomicrobial gut symbiont with high potential for hemicellulose degradation.

## Graphical abstract

**Figure:**
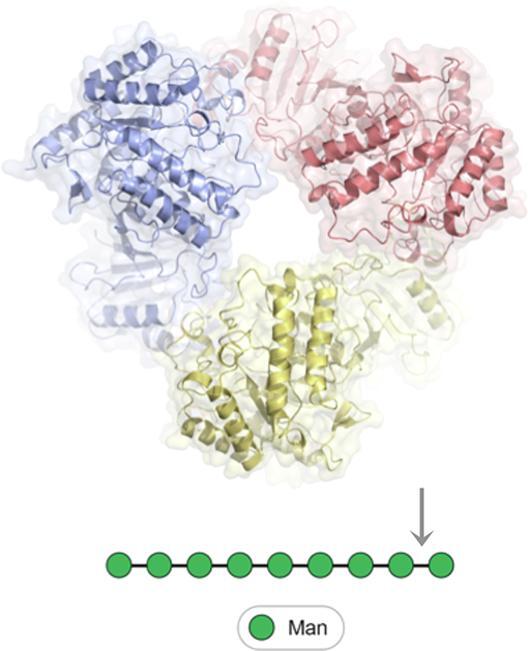

## Introduction

Wood-feeding termites are known to cause substantial damage to man-made wood-based constructions, buildings and trees, with an estimated economical loss of 15 to 40 billion USD per year (Govorushko, 2019). Notwithstanding their destructive potential, termites are attracting considerable interest for biomass processing due to their gut microbiomes that produce a rich variety of wood-degrading enzymes (Peterson and Scharf, 2016). For a wood-based diet, termites rely on the obligate symbiotic relationship with specialized microbes that inhabit the saliva and digestive tract.

The well studied and highly invasive Eastern Mediterranean termite *Reticulitermes flavipes* (Kollar) is considered to be the most harmful and destructive termite, and responsible for causing considerable damage and economical loss (Su and Scheffrahn, 1990). The efficient saccharification of wood by *R. flavipes* is enabled by symbionts that evolved to target specifically recalcitrant lignocellulose and hemicellulose constituents, and the usefulness of this termite for processing of lignocellulosic biomass has been reported (Karl and Scharf, 2015, Rajarapu et al., 2015, Rajarapu and Scharf, 2017). The hindgut lumen symbiota of *R. flavipes* has been characterized (Boucias et al., 2013), and the microbiome was found to include mainly bacteria (nine major phyla, 4761 species-level phylotypes), with a minor population of archaea (phyla Korarchaeota and Euryarchaeota).

One of the bacterial genomes identified in the *R. flavipes* hindgut is that of the gram-negative *Opitutaceae* bacterium strain TAV5 of the Verrucomicrobia phylum (Kotak et al., 2015). Verrucomicrobial members are widespread, but the relative abundance of verrucomicrobial communities differs (Nixon et al., 2019) (2% in the *R. flavipes* hindgut (Boucias et al., 2013). Despite their low relative abundance, these bacteria play an important role in carbon cycling, and their genomes carry complete sets of genes for degradation of a range of polysaccharides, including hemicelluloses (Nixon et al., 2019, Martinez-Garcia et al., 2012, Herlemann et al., 2013, Cardman et al., 2014). *Opitutaceae* bacterium strain TAV5 has a comparably large genome among known verrucomicrobial members, approximately 7.4 Mbp, including predicted enzyme activities for degradation of wood-based components (Kotak et al., 2015, Nixon et al., 2019). For the achievement of improved efficiency of biomass processing, considerable efforts have been devoted to identify microbial enzymes capable of degrading the recalcitrant fractions of crystalline cellulose in plant-based biomass, while less progress has been made to identify specialist enzymes for the conversion of the hemicellulose fraction, which depending on the source of wood can amount to about 40% of the dry weight (Fan et al., 1982). Hemicellulose components of plant cell walls include, *e.g*., xylan, glucan, xyloglucan, glucuronoxylan, galactoglucomannan, glucomannan, glucuronomannan, arabinoxylan, arabinogalactan, arabinoglucuronoxylan.

*β*-mannosidases (EC 3.2.1.25) are *exo*-acting enzymes that catalyze the successive release of D-mannose from the non-reducing end of various *β*-1,4-linked mannans and mannooligosaccharides, and cooperate with endolytic *β*-mannanases. Mannan-degrading enzymes are widely used in various industrial processes such as the pulp and paper treatment, coffee extraction, oil and gas drilling, biofuel, food and animal feed (Malgas et al., 2015). In addition, *β*-mannosidases are used in the biosynthesis of oligosaccharides and alkyl *β*-mannosides for medical and other purposes (Eneyskaya et al., 2009, Zhang et al., 2009, Zhou et al., 2014). With the exception of the *β*-mannosidases from the human gut symbionts *Roseburia intenstinalis* (*Ri*GH113) and *Bacteroides salyersiae* (*Bs*164), which belong to CAZy (www.cazy.org (Lombard et al., 2014) glycoside hydrolase families 113 and 164, respectively, *exo*-*β*-mannosidases are found in the GH families GH1, GH2 and GH5 (Shallom and Shoham, 2003). Here, we report the biochemical characterization and three-dimensional structure of the GH5 *exo*-*β*-1,4-mannosidase *Op5*Man5 from the *Opitutaceae* bacterium strain TAV5 present in the hindgut of the wood-feeding termite *R. flavipes*.

## Results and discussion

### *Op5*Man5 sequence and biochemical properties

The gene coding for *Op5*Man5 (locus 10225; UniProt W0J1H8) has been annotated as a putative *endo*-*β*-mannanase (EC 3.2.1.78) that belongs to the GH5 family of glycoside hydrolases (www.cazy.org (Lombard et al., 2014). The primary structure comprises 594 amino acids, lacks a secretion signal, and corresponds to a theoretical isoelectric point of 5.6 and molecular weight of ∼ 66 kDa. According to SDS-PAGE analysis, the molecular mass is ∼ 68 kDa for the recombinant *Op5*Man5 produced in *Escherichia coli*, and 66824 g/mol according to electrospray ionization mass spectrometry (ESI-MS) (Figure S1). The ESI-MS-derived molecular mass agrees well with the theoretical molecular weight for full-length *Op5*Man5.

Analysis of the *Op5*Man5 oligomeric state was performed using size-exclusion chromatography (SEC) using a set of reference proteins with known molecular weights to calculate a standard curve (Figure S2(A)). At pH values 5.5–6.5 (Figure S2(B-D)), the SEC profile is monodisperse corresponding to the homotrimer assembly with a molecular weight of ∼214 kDa according to the standard curve. At pH 7.5 (Figure S2(E)), the SEC profile becomes bidisperse with the homotrimer species appearing in equilibrium with a species of twice its molecular weight (∼434 kDa), presumably corresponding to a homohexamer. Increasing the pH further (pH 8.0–8.5) shows a gradual disappearance of the presumed hexamer with a concomitant increase of the homotrimer (Figure S2(F-G)). Thus, hexameric *Op5*Man5 appears to exist only within a relatively narrow pH range around 7.5. Below pH 5.5, the enzyme precipitates (data not shown), a process that starts already at pH 5.5, as indicated by the lower amount of *Op5*Man5 at pH 5.5 (Figure S2(B)). Following the SEC analysis, we performed chemical cross-linking analysis, which confirmed a major homotrimeric species, and a minor hexameric assembly (Figure S3).

## Substrate screening and kinetic characterization

**Fig. 1:**
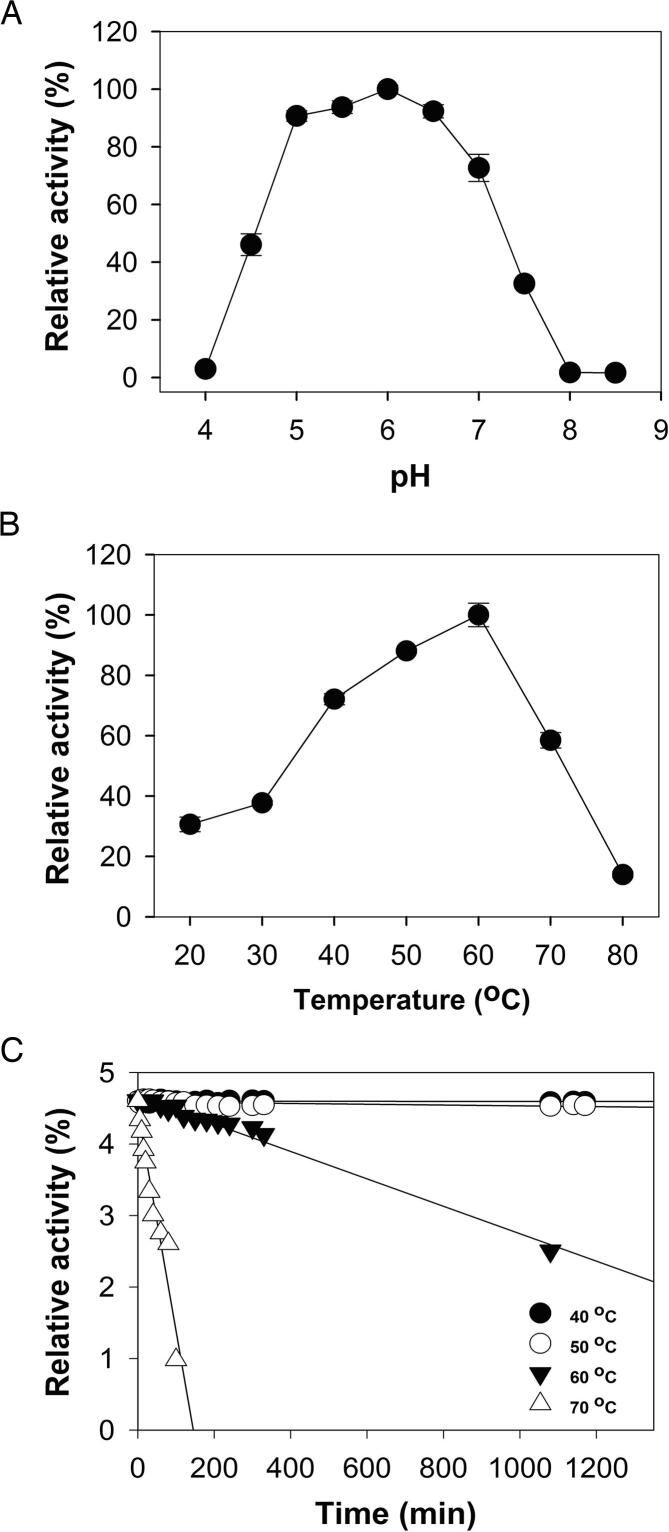
Activity optima and decay as a function of pH and temperature. Effect of (A) pH and (B) temperature on the activity profile of *Op5*Man5 with *p*NP-*β*Man as substrate. (C) Thermal inactivation of *Op5*Man5. The enzyme was incubated in vials at different temperatures. Samples (10 μl) were withdrawn at the indicated time points and tested for enzyme activity at room temperature by using *p*NP-*β*Man as substrate. All experiments were performed at pH 6.0.

**Fig. 2:**
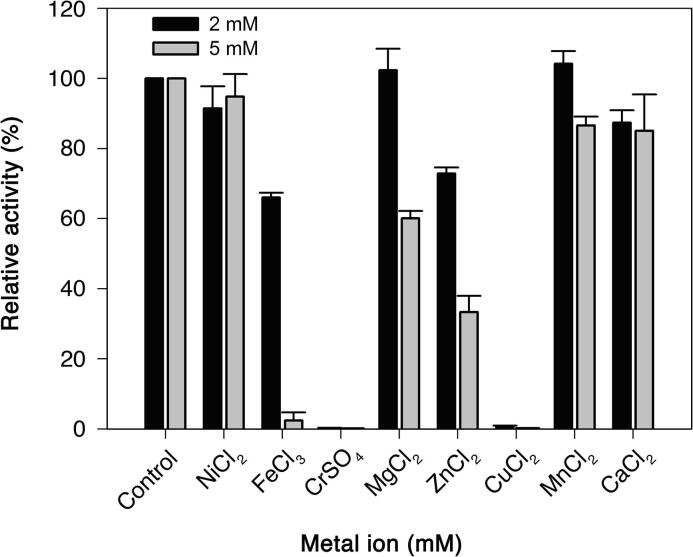
Effect of different metal ions on activity. The influence of metal ions (2 and 5 mM) on *Op5*Man5 activity using *p*NP-*β*Man as substrate at pH 6.0.

Of the aryl glycosides tested, only *p*NP-*β*-D-mannopyranoside (*p*NP-*β*Man) served as substrate for recombinant *Op5*Man5 (Table 1), which establishes the specificity for *β*-1,4-mannosidic bonds. The apparent *K*_m_ and *k*_cat_ values for *p*NP-*β*Man were 1.0 mM and 238.9 s^−1^, respectively, and the specificity constant (*k*_cat_/*K*_m_) of 238.9 mM^−1^ s^−1^ (Table 2). The pH optimum of activity for *p*NP-*β*Man as the substrate was 6.0 (Fig. 1(A)), at which the temperature (*T*) optimum was 60 °C (Fig. 1(B)). Thermal inactivation (preincubation at *T* = 40, 50, 60, 70 °C *versus t*) showed that the activity was decreasing rapidly above 60 °C (Fig. 1(C)). Based on the inactivation profile, pH 6.0 and 40 °C were chosen as the standard assay conditions. The catalytic dependency of EDTA-treated *Op5*Man5 on various metal ions was investigated, and no significant enhancement of activity was observed for the metal ions tested, however, several metal ions showed an inhibitory effect at 5 mM concentration, i.e.*,* Fe^2+^, Mg^2+^ and Zn^2+^ (Fig. 2).

**Table 1:** Substrate specificity of *Op5*Man5.

Substrate	Specific activity (U⋅mg^−1^)^a^	Relative activity (%)^b^
*Aryl glycosides*^c^^,^^d^		
*p*NP-*β*Man	92 ± 10.4	100
*p*NP-*α*Man	ND	ND
*p*NP*β*-Xyl	ND	ND
*p*NP-*β*Fuc	ND	ND
*p*NP-*α*Fuc	ND	ND
*p*NP-*β*Gal	ND	ND
*p*NP-*β*Glc	ND	ND
*p*NP-*β*Cel	ND	ND
*Mannooligosaccharides*^c^^,^^d^		
M2	1.3 ± 0.2	50
M3	1.4 ± 0.2	53
M4	1.7 ± 0.3	67
M5	2.2 ± 0.1	85
M6	2.6 ± 0.2	100
*Polysaccharides*^c^^,^^d^		
Ivory nut mannan	0.29 ± 0.07	100
Konjac glucomannan	0.18 ± 0.02	63
Galactomannan	ND	ND
LGB	ND	ND
Xylan	ND	ND
Barley β-glucans	ND	ND
CMC	ND	ND

a The specific activity on aryl glycosides is expressed as the amount of enzyme (in mg) releasing 1 μmol⋅min^−1^ pNP. The specific activity on mannooligo- and polysaccharides was measured using the GOD-POD assay and is expressed as the amount of enzyme (in mg) releasing 1 μmol⋅min^−1^ glucose.

b The relative activity was calculated from the specific activity of each substrate with the most preferred substrate set to 100%.

c Errors of experimental data are represented as mean ± standard deviation from triplicate experiments.

d ND, not detected

**Table 2:** Kinetic parameters.

Substrate	*K*_m_ (mM)	*k*_cat_ (s^−1^)	*k*_cat_ / *K*_m_ (s^−1^·mM^−1^)
*p*NP-*β*Man	1.0 ± 0.13	238.9 ± 7.7	238.9 ± 7.7
M3	–	–	2.7 ± 0.4
M4	–	–	7.8 ± 1.0
M5	–	–	14.7 ± 2.1
M6	–	–	18.9 ± 1.4

For *β*-1,4-linked mannooligosaccharides (MOSs) with a degree of polymerization (DP) of 2 to 6 (M2, M3, M4, M5, M6) the specific activity increased with increasing DP with the lowest being 1.3 U mg^−1^ for M2, and the highest value of 2.6 U mg^−1^ observed for M6 (Table 1). For the polysaccharides, activity was only observed for ivory nut mannan (0.29 U mg^−1^) and Konjac glucomannan (0.18 U mg^−1^). The lower activity on glucomannan indicates that the presence of glucose in the backbone of this type of mannan negatively affects hydrolysis compared with a pure *β*-mannan backbone. Michaelis-Menten kinetics for MOSs of DP 3 to 6 (pH 6.0, *T* = 40 °C, *t* = 0–12 h) shows that the specific catalytic efficiency increases with DP, with the highest *k*_cat_/*K*_m_ value observed for M6 (Table 2). The catalytically active residues Glu156 and Glu281 were inferred from sequence comparisons with related GH5 enzymes. The single replacement E156Q showed no activity against MOSs, but weak activity against *p*NP-*β*Man. The single replacement E281Q and the double mutant E156Q/E281Q displayed no activity against either *p*NP-*β*Man or MOSs, which supports the assignment of Glu281 as the catalytic nucleophile.

Activity towards *p*NP-*β*Man has been reported for a number of *exo*-*β*-1,4-mannosidases (Table S1). Comparing the kinetic parameters for *p*NP-*β*Man as substrate shows that the catalytic performance varies considerably across enzymes. *Op5*Man5 displays a similar K_m_ value, but higher specificity constant compared to the bacterial GH5_7 *exo*-*β*-1,4-mannosidase *Cm*Man5A from *Cellvibrio mixtus* (Dias et al., 2004), the fungal GH5 *exo*-*β*-1,4-mannosidase *Lr*Man5B from *Lichtheimia ramose* (Xie et al., 2019), the mammalian GH2 *exo*-*β*-1,4-mannosidase ManbA from mouse (Gytz et al., 2019), and the archaeal GH1 *exo*-*β*-1,4-mannosidase TkβGly from *Thermococcus kodakarensis* (Hwa et al., 2015). Only a few enzymes have higher specificity constant than that of *Op5*Man5, and with the exception of the GH5_19 member *Tth*Man5 from *Pseudothermotoga thermarum* (Shi et al., 2013), they are all bacterial GH2 enzymes: *Bt*Man2A from *Bacteroides thetaiotaomicron* (Tailford et al., 2007); *Cf*Man2A from *Cellulomonas fimi* (Stoll et al., 1999); *Dt*Man from *Dictyoglomus thermophilum* (Guillotin et al., 2017), Man2S27 from *Streptomyces* (Shi et al., 2011), and the GM-1 isoenzyme from *Phlebia radiate* (Prendecka et al., 2007).

**Fig. 3:**
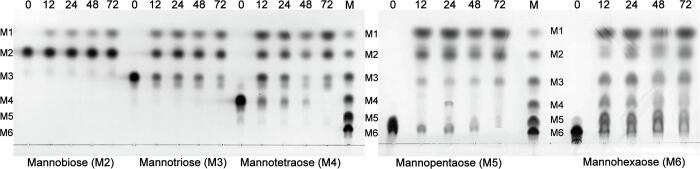
TLC analysis. Time-dependent hydrolysis of MOSs with DP 2–6.

**Fig. 4:**
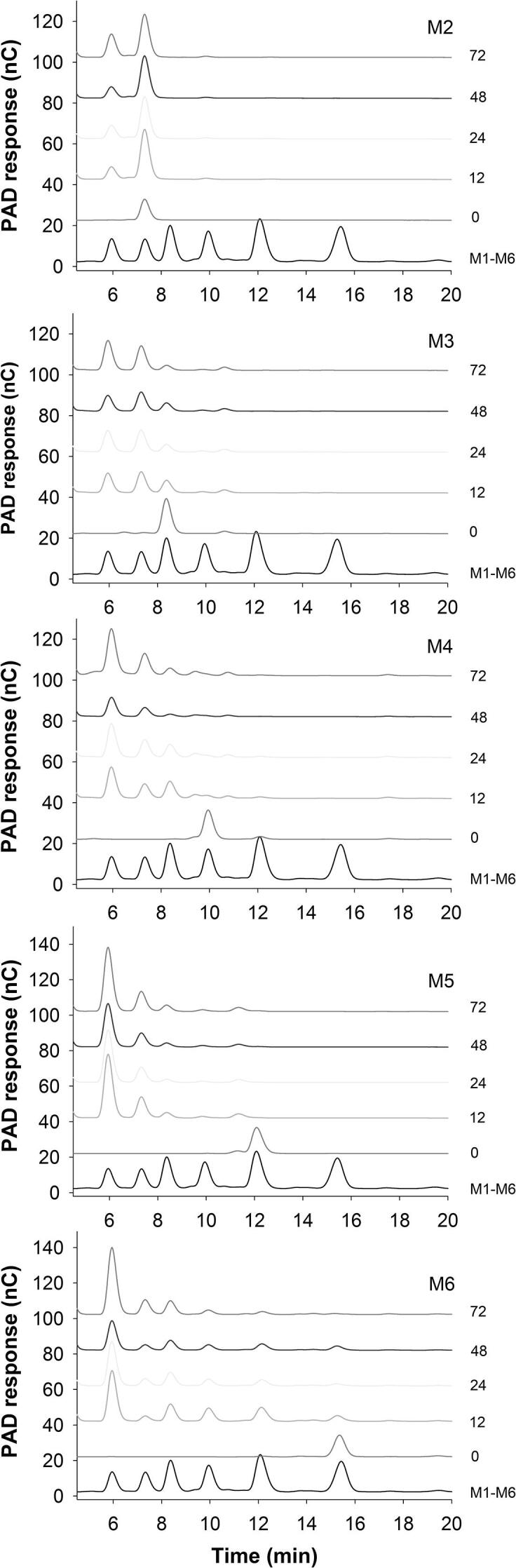
HPAEC-PAD product analysis for hydrolysis of MOS (DP 2–6) by *Op5*Man5. The black curve represents MOS standards with DP 1–6 (left to right).

The reaction products from hydrolysis of various MOSs were studied by TLC and HPLC (Fig. 3 and Fig. 4). *Op5*Man5 hydrolyzed the *β*-1,4-linkages in M3, M4, M5 and M6 to produce M1 and M2 as the major products. M1 was successively released from all MOSs, supporting an *exo*-type action. An increasing efficiency of conversion to M1 was observed with increasing substrate DP. Whereas M6 was almost completely converted to M1 after 72 h, hydrolysis of M2 and M3 was considerably slower.

Similar stepwise hydrolysis patterns of *β*-1,4-linked MOSs have been reported for several *exo*-*β*-1,4-mannosidases, including enzymes belonging to families GH5 (Zhou et al., 2014, Dias et al., 2004, Li et al., 2015, Reichenbach et al., 2018), GH2 (Zhang et al., 2009, Tailford et al., 2007, Zahura et al., 2012) and GH1 (Xu et al., 2004), and non-classified *exo*-*β*-1,4-mannosidases (McCleary, 1983, Akino et al., 1988, Gübitz et al., 1996, Andreotti et al., 2005). Examples of enzymes that, like *Op5*Man5, release mannose from both MOSs and high-molecular weight substrates include *Cm*Man5A (from ivory nut mannan and 1,4-*β*-mannan with a DP of 15 (Dias et al., 2004), *Ac*Man5 (Li et al., 2015) and *Ak*Mnsd (Zahura et al., 2012) (from linear *β*-mannan), *Lr*Man5B (from locust bean gum galactomannan (Xie et al., 2019), *Bt*Man2A (from linear mannan, but also small amounts from galactomannan or glucomannan (Tailford et al., 2007), and the *β*-mannosidase from *Athelia rolfsii*, which was reported to completely depolymerize all mannans, gluco- and galactomannans tested (Gübitz et al., 1996).

Retaining glycoside hydrolases have been shown to catalyze transglycosylation reactions, and to evaluate the ability of *Op5*Man5 to catalyze transglycosylation, we analyzed transfer reactions using M2 as donor substrate and alcohols as acceptors. TLC analysis of the products formed as a results of transglycosylation reactions shows that *Op5*Man5 catalyzed hydrolysis and transglycosylation reactions simultaneously (Figure S4). MOSs with DP > 2, *i.e*., 3, 4 and minor amounts of 6 were observed with methanol, ethanol, 1-propanol, or hexanol as acceptors. In addition, substantial amounts of the corresponding alkyl *β*-mannosides were detected in the TLC analysis in presence of the tested alcohols.

It is common for glycoside hydrolases to be activated by an alcohol and to catalyze the production of alkyl glycosides, which is largely attributed to transglycosylation (Lundemo et al., 2013, Morrill et al., 2018), and there are several examples in the literature of transglycosylation by *β*-mannosidases and *β*-mannanases with alcohols as acceptors (Zhang et al., 2009, Morrill et al., 2018, Kurakake and Komaki, 2001, Rosengren et al., 2014). The acceptor specificity of *Op5*Man5 suggests that it is attractive for synthesis of oligosaccharides and alkyl glycosides, which are compounds that can be used in industrial applications for the production of food additives, animal feed, detergents, and personal care products. *β*-mannosidases reported to have transglycosylation activity are also indicated in Table S1.

## Structure of *Op5*Man5

Crystals of *Op5*Man5 grew from a solution containing phosphorus citrate (pH 4.2), 30% (v/v) ethanol, 5% (w/v) polyethylene glycol 1000, and were subjected to soaking with 1 mM UO_2_(NO3)_2_. *Op5*Man5 crystallized in space group *P*2_1_2_1_2_1_ with one homotrimer per asymmetric unit and diffracted to 2.2 Å resolution (Table S2). The rationale for adding the uranyl compound was to obtain experimental phases by isomorphous replacement, however, no significant signal was obtained. A large number of alternative heavy atoms were tested, but despite extensive screening, no crystals were sufficiently derivatized to allow heavy-atom substructure determination, which was likely due to the large protein content of the asymmetric unit (200 kDa), a relatively high solvent content (65%), and overall poor resolution of heavy-atom data sets.

We also performed structure prediction using the fold-recognition server *Phyre2* (Kelley et al., 2015). The only high confidence hits were homotrimeric GH42 *β*-galactosidases (e.g., PDB codes 1KWG, 5E9A, 3TTS, 4UZS, 4OJY). Based on this information, extensive attempts were made to obtain preliminary phases by molecular replacement, but without success. All else having failed, an attempt was made to estimate phases by single particle electron cryo-microscopy (cryo-EM). Images were recorded and processed for *Op5*Man5 at pH 7.5 to yield a 3.7 Å resolution Coulomb potential map that allowed a partial model to be built. The partial model was then used to phase the 2.2-Å X-ray diffraction data using molecular replacement to determine the crystal structure of *Op5*Man5. Although only SEC fractions at pH 7.5 (Figure S2(E)) containing the trimeric species were pooled for the cryo-EM experiment, the EM micrographs show clear evidence of larger particles presumably corresponding to a hexamer or heptamer (Figure S5). Due to the low number of large particles, only the homotrimeric state was considered for image processing. Determination of the precise identity and oligomeric state of the larger assembly would require EM data presenting a significant increase of the number of large particles, which is currently not available.

**Fig. 5:**
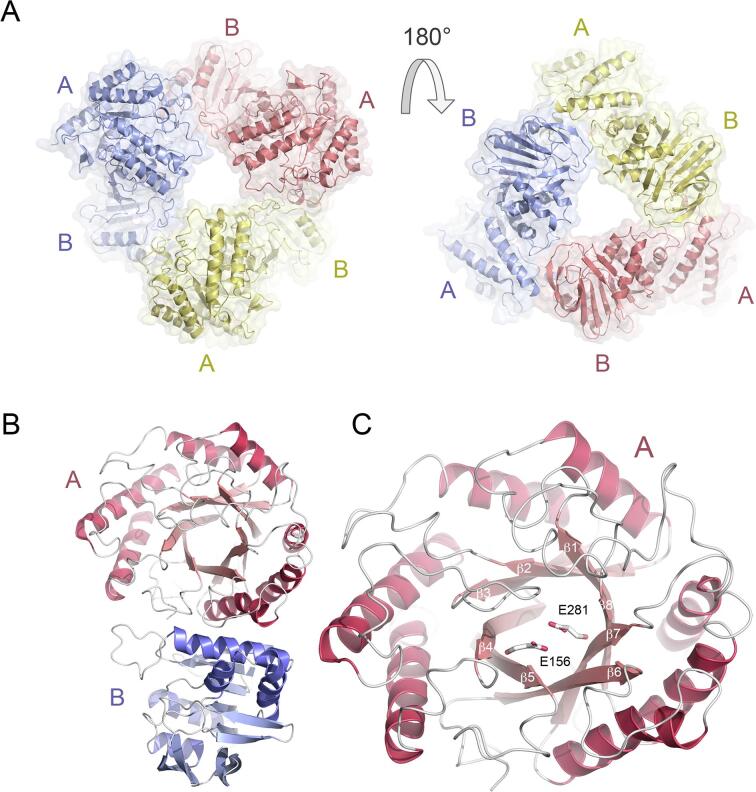
Overall fold of *Op5*Man5. (A) The homotrimer shown from two sides, front and back. Each modular monomer consists of two domains, A and B. (B) The modular monomer with the catalytic domain A (red) and trimerization domain B (blue). (C) Enlarged picture of domain A that forms a TIM barrel with the catalytic residues Glu156 and Glu281 positioned in β strands 4 and 7, respectively. (For interpretation of the references to colour in this figure legend, the reader is referred to the web version of this article.)

*Op5*Man5 is a homotrimer consisting of three modular monomers (Fig. 5(A)) arranged as described for GH42 *β*-galactosidases. The modular monomer structure (Fig. 5(B) and Figure S4(A)) contains an N-terminal catalytic (β/α)_8_ (TIM) barrel that belongs to clan GH-A with a sequence placed in the GH5 family of retaining glycoside hydrolases (domain A; residues 2–364). The A-domain is followed by a Rossmann-like β-sandwich domain (domain B; residues 367–586). The two catalytic glutamate residues 156 and 281 are situated in β strands 4 and 7 of the TIM barrel, respectively (Fig. 5(C)). *Op5*Man5 lacks the small C-terminal Greek key β-barrel domain typical for GH42 *β*-galactosidases.

**Fig. 6:**
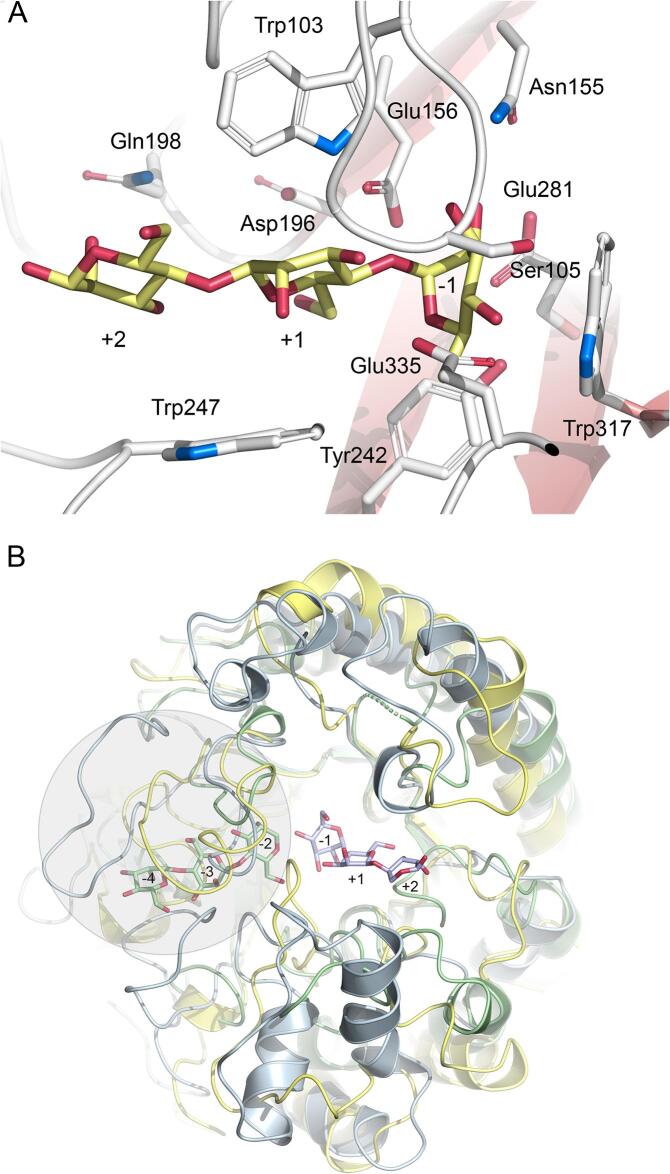
The active site in *Op5*Man5. (A) *Op5*Man5 with a mannotriose molecule modeled in the active site with the three mannose units occupying subsites −1, +1 and + 2. Relevant side chains that are suitably positioned to interact with the substrate are shown. (B) Overlay of the TIM barrel in *Op5*Man5 (yellow), *Rm*Man5B (light blue; PDB code 4LYQ) and *Tf*Man (green; PDB code 3MAN). The mannotriose molecule in the *exo*-*β*-1,4-mannosidase *Rm*Man5B occupies subsites –1 to + 2, and in the *endo*-*β*-mannanase *Tf*Man, mannotriose is bound at subsites –2 to –4. The loops forming the steric block that closes off the negative subsites in the *exo*-acting enzymes *Op5*Man5 and *Rm*Man5B are indicated by a gray circle. (For interpretation of the references to colour in this figure legend, the reader is referred to the web version of this article.)

As expected for an *exo*-acting *β*-mannosidase, the active site in *Op5*Man5 is located in a pocket with the two catalytic glutamate residues (Glu156 and Glu281) buried roughly 10 Å below the surface of domain A. All three active sites are accessed from the central void of the homotrimer. By manually placing mannooligosaccharides in the active site of the A-domain, possible binding sites for three mannose units are readily identified (Fig. 6(A)). The innermost binding site, subsite –1, includes Glu281, Trp317, Glu335, Ser105, Tyr242, and Asn155. Glu156 is positioned between subsites –1 and + 1. Trp103, Gly106 and possibly Asp196 are suitably positioned to interact with a mannose unit in subsite + 1, Trp247 can act as a platform roughly below the second glycosidic bond between subsites + 1 and + 2 in a docked mannotriose molecule. Gln198 is positioned to interact with a mannose unit in subsite + 2. The observed increase in *k*_cat_/*K*_m_ from 2.2 mM^–1^s^−1^ for M3 to 18.9 mM^–1^s^−1^ for M6 (Table 2) suggests that there are binding sites beyond the subsite + 2 that contribute to binding and hydrolysis efficiency. Residues with longer side chains at the interface between domains A and B could possibly provide a framework for additional binding sites, e.g., Glu405, Arg407, Arg411 and Arg517.

Two loop regions in *Op5*Man5 (residues 103–108 and 320–339) cooperate to form a steric block that closes off the active site to form a pocket (Fig. 6(B)). This type of steric block was first described for *Cm*Man5A (Dias et al., 2004), and later also for the fungal GH5_7 enzyme *Rm*Man5B from *Rhizomucor miehei* (PDB code 4LYQ (Zhou et al., 2014). In *Cm*Man5A and *Rm*Man5B, a continuous loop region forms the steric block (residues 379–410 in *Cm*Man5A and residues 357–386 in *Rm*Man5B), rather than two separate loops. The steric block has been assigned a structural role in converting a GH5-type *endo*-mannanase into an *exo*-acting mannosidase (Zhou et al., 2014, Dias et al., 2004). The trajectory of a mannan chain that occupies the blocked negative subsites in an *endo*-acting GH5 mannanase is exemplified by the GH5_8 *β*-mannanase *Tf*Man from *Thermobifida fusca* in complex with mannotriose where the three mannose units occupy subsites –2, –3 and –4 (PDB code 3MAN (Hilge et al., 1998), which are disabled by the steric block in the *exo*-acting *Op5*Man5, *Cm*Man5A and *Rm*Man5B (Fig. 6(B)).

The B-domain is referred to as a trimerization domain in homotrimeric GH42 β-galactosidases. Analyzing the *Op5*Man5 structure using PISA gives a CSS (complexation significance score) of 1.0 for a homotrimer, which is expected for a natural oligomerization state. Pairwise monomer interface areas range between 1112 and 1154 Å^2^ with a solvation free energy gain of −8.7 to −10.0 kcal/mol. There are no salt bridges formed between individual monomers, and trimer stabilization is partly provided through 17 hydrogen bonds between the B-domain and the A-domain of a neighboring monomer. Six regions in the trimerization domain provide trimer interactions: 399–402, 453–458, 477–484, 491–499, 522–526 and 517–519, all of which make contact with the A domain of a neighboring monomer. There are no direct interactions between B domains of individual monomers in the trimer.

Two regions in *Op5*Man5 map have weak, unresolved electron density, and were therefore not modeled: a flexible region in domain A between β5 and β6, including the region expected to form α5 (residues 213–221); and the linker region that connects domains A and B (residues 368–381). Additionally, the last seven residues of the C-terminus appear disordered. The missing regions are located at the surface, on the side of the TIM barrel opposite to the active-site entrance, and neither is therefore expected to influence substrate binding or catalysis. However, it cannot be excluded that these flexible regions may play a role in formation of the high-order oligomer that is predicted to be a hexamer or heptamer.

## Structural and functional similarity to other GH enzymes

Despite insignificant sequence identity (12–17%) to sequences of GH42 *β*-galactosidases, the A and B domains of the GH5 member *Op5*Man5 and have the same topology and overall structure as the homotrimeric GH42 *β*-galactosidases (Figure S6 and Figure S7). There are several structures of GH42 *β*-galactosidases, and *Ra*BGal from *Rhanella* sp. R3 (PDB code 5E9A (Fan et al., 2016) was chosen for the purpose of structural comparison. Root-mean-square deviation values for aligned Cα atoms for the individual domains of *Op5*Man5 and *Ra*BGal are 2.2 Å for 269 (of 349) aligned Cα positions (16.0% sequence identity) for domain A; and 2.6 Å for 165 (of 204) aligned Cα positions (16.4% sequence identity) for domain B. While the overall structure of the *Op5*Man5 homotrimer is similar to that of the GH42 *β*-galactosidases, the active sites are more diverse. Besides the trimeric *β*-galactosidases, the only other enzymatic activity found in family GH42 is the *α*-L-arabinopyranosidase from *Bifidobacterium animalis* (*Bl*Arap42B; PDB code 5XB7 (Viborg et al., 2017), also a homotrimer, and the enzyme displays structural similarity to *Op5*Man5 comparable to that of the GH42 *β*-galactosidases: 2.2 Å for 261 (of 349) aligned Cα positions (22.5% sequence identity) for domain A; and 2.4 Å for 165 (of 204) aligned Cα positions (15.2% sequence identity) for domain B.

Additionally, the *exo*-*β*-1,4-mannosidase *Bs*164 (PDB code 6T7G (Armstrong and Davies, 2020) from the human gut symbiont *Bacteroides salyersiae* is a homotrimer with striking similarity to the GH42 *β*-galactosidases. *Bs*164 has been classified as a member of family GH164, but regardless, the enzyme features the topology and tertiary structure of the GH42 *β*-galactosidases (Figure S6 and Figure S7). As in the case of *Ra*BGal, the sequence identity shared between *Bs*164 and *Op5*Man5 is low, and structural comparison gives an overall r.m.s.d. value for the monomer of 2.5 Å for 424 (of 553) aligned Cα positions (14.2% sequence identity). For domain A, the r.m.s.d. value is 2.2 Å for 257 (of 349) aligned Cα positions (14.4% sequence identity), and for domain B, 2.3 Å for 168 (of 204) aligned Cα positions (16.1% sequence identity). It should be noted that the individual domains in the three enzymes show minor rotations relative each other.

The structure of the *exo*-*β*-mannosidase *Ca*Man5 from *Cutibacterium acnes* has been reported (PDB code 6GVB (Reichenbach et al., 2018). Like *Op5*Man5, this enzyme belongs to GH5, but is active as a homodimer and has been assigned subfamily 18 of the GH5 sequences. *Ca*Man5 hydrolyzes the *β*-1,4 mannosyl linkage in Man-*β*-1,4-GlcNAc of the *N*-glycan core. The *Ca*Man5 monomer is not modular in architecture, and includes only the catalytic TIM-barrel domain. Structural comparison of the TIM-barrel domain *Op5*Man5 and *Ca*Man5 returns an r.m.s.d. value of 1.9 Å for 333 aligned Cα positions corresponding to a sequence identity of 22.8%. A similar r.m.s.d. value (1.8 Å for 349 aligned Cα positions; 24.5% sequence identity) is found for the homodimeric *exo*-*β*-mannosidase *Bl*Man5B from the human gut symbiont *Bifidobacterium longum* (PDB code 6MPA (Cordeiro et al., 2019), which is functionally and structurally closely related to *Ca*Man5.

Indeed, the low sequence similarity to structurally characterized enzymes makes a detailed comparison difficult. By considering only the catalytic TIM-barrel domain, a reasonable structure-based sequence alignment can be generated for the GH5 *exo*-*β*-1,4-mannosidase with known structure, *i.e*., *Op5*Man5, *Ca*Man5, *Bl*Man5B, *Cm*Man5A and *Rm*Man5B (Figure S8). Including the GH2, GH1 and GH164 enzymes was not considered meaningful because of too pronounced structural differences. The sequence alignment shown in Figure S8 was created by performing a structural superimposition of the TIM-barrel domains to align equivalent residues spatially. This results in a sequence identity of 24% between *Op5*Man5 and the best matching homologs (*Ca*Man5 and *Bl*Man5B), and lower percentages for *Cm*Man5A (16.8%) and *Rm*Man5B (16.4%). Thus, even the catalytic TIM-barrel, which is the most similar region of these GH5 *exo*-*β*-1,4-mannosidase, show very low similarity. Considering that all of these enzymes display *exo*-*β*-1,4-mannosidase, there are very few conserved residues in the active site, and identities across all five enzymes are limited to the catalytic glutamate residues Glu156 and Glu281, Arg58, Asn155, His240 and Trp317 (*Op5*Man5 numbering). The homodimeric *exo*-*β*-1,4-mannosidases *Ca*Man5 and *Bl*Man5B are active on the *β*-1,4-linkage in Man-GlcNAc of the chitobiose core of *N*-glycans. Despite the overall structural differences between *Op5*Man5 and these enzymes, their active sites show higher resemblance to that of *Op5*Man5 than do *Cm*Man5A and *Rm*Man5B. The entrance of the substrate-binding pocket in *Op5*Man5, *Ca*Man5, and *Bl*Man5B shows a similar shape, that differs from that of *Cm*Man5A and *Rm*Man5B (Figure S8).

Using BLASTP (https://blast.ncbi.nlm.nih.gov/Blast.cgi) to search the database of non-redundant protein sequences for sequences similar to that of *Op5*Man5 returned only sequences of uncharacterized GH enzymes, many of which contain the *β*-galactosidase trimerization domain. As expected, the closest related sequences were from the *Opitutaceae* family of the Verrucomicrobia phylum (Figure S9). Possible homologs of *Op5*Man5 are also found in *Streptomyces* and *Bacillus* species, but with considerably lower sequence similarities.

## GH enzymes in *Opitutaceae* bacterium strain TAV5

The genome of *Opitutaceae* bacterium strain TAV5 comprises 7.4 Mbp and includes a large number of genes that are relevant for the degradation of polysaccharides, glycans and glycoconjugates. Of these, 257 genes have been classified in the CAZy database as glycoside hydrolases, representing 49 different GH families. Of the GH genes, 44% (114 genes) belong to families dominated by hemicellulose-degrading activities (GH39, GH148, GH78, GH2, GH5, GH38 and GH110) with predicted activity on *β*-1,3-glucans, *β-*xylose-, arabinose-, rhamnose- and *α*- or *β-*mannose-containing compounds (Figure S10). The *β*-xylosidase family GH39 is by far the most well-represented GH family in the *Opitutaceae* bacterium strain TAV5 genome with 47 predicted GH39 genes (mainly *β-*xylosidases, *α*-L-arabinofuranosidases and *β-*galactosidases). The second and third most populated families are GH148 (*β*-1,3-glucanses) and GH78 (*α*-L-rhamnosidases) with 17 and 14 genes, respectively; followed by GH5 represented by 9 genes.

## Conclusions

In this work we have reported on the characterization of a new hemicellulose-degrading enzyme, *Op5*Man5, from the bacterial symbiont *Opitutaceae* bacterium strain TAV5 thriving in the hindgut of the notorious termite pest *R. flavipes*. We used single particle cryo-EM to generate an initial 3.7-Å model molecular model for further phasing of synchrotron intensity data at 2.2 Å resolution using molecular replacement. The structure reveals that *Op5*Man5 forms a 200-kDa homotrimer, which represents the first example of a GH5 enzyme with a GH42-*β*-galactosidase-type homotrimeric assembly. Interestingly, within a narrow range around pH 7.5, the homotrimer is observed to be in equilibrium with a hexameric or heptameric state, which could also be observed in small numbers in the cryo-EM micrographs. While the opit5_10225 gene had been annotated as an *endo*-*β*-mannanase in the sequence databases, our work establishes that the enzyme is an *exo*-*β*-1,4-mannosidase with high specificity for the terminal *β*-1,4-mannosidic linkages in mannooligosaccharides and linear, mainly unsubstituted, *β*-mannans such as ivory nut mannan. Furthermore, in the presence of alcohols, *Op5*Man5 catalyzes simultaneous hydrolysis and transglycosylation reactions of mannobiose into mannooligosaccharides and alkyl *β*-mannosides, compounds that are attractive for the production of food additives, animal feed, detergents, and personal care products. The abundance of GH39, GH148, GH78, and GH5 genes in the genome of *Opitutaceae* bacterium strain TAV5 strongly suggests that this verrucomicrobial member is specialized towards degradation of the hemicellulose fractions of wood-based termite feed.

## Materials and methods

### Materials

The 4-nitrophenyl (*p*-nitrophenyl, *p*NP) substrates *p*NP-*β*-D-mannopyranoside (*p*NP-*β*Man), *p*NP-*β*-D-glucopyranoside (*p*NP-*β*Glc), *p*NP-*β*-cellobiose (*p*NP-*β*Cel), *p*NP-*α*-D-glucopyranoside (*p*NP-*α*Glc), *p*NP-*β*-D-xylopyranoside (*p*NP*β*-Xyl), *p*NP-*β*-D-fucopyranoside, (*p*NP-*β*Fuc), *p*NP-*α*-L-fucopyranoside (*p*NP-*α*Fuc) and *p*NP-*β*-D-galactopyranoside (*p*NP-*β*Gal) were purchased from Sigma Chemical Co. (St. Louis, MO). Locust bean gum (LBG, carob seed galactomannan), birchwood xylan, carboxy methylcellulose (CMC, low viscosity), β-D-1-thiogalactopyranoside (IPTG), Tris (2-carboxyethyl) phosphine hydrochloride (TCEP) were from Sigma. Galactomannan (locust bean gum), Konjac glucomannan (low viscosity, mannose:glucose 60:40), mannan (ivory nut, 99% mannose), and mannooligosaccharides: mannobiose (M2), mannotriose (M3), mannotetraose (M4), mannopentaose (M5), and mannohexaose (M6); were purchased from Megazyme (Bray, Ireland).

### Gene cloning and site-directed mutagenesis

*Op5*Man5 full length DNA (GenBank accession no. AHF90519) was synthesized from GenScript. The gene was subcloned into the ligation-independent cloning (LIC) vector *p*NIC-CH2 adding a C-terminal, non-cleavable *hexa*-histidine tag (https://psf.ki.se) using the following forward and reverse primers:

LIC_fwd: 5′- AGAGGAGATAATTAATGACCATCGATTCTTCCGGTTAC-3′

LIC_rev: 5′- AATGGTGGTGATGATGGTGCGCACGCGCGACCCCTTCGG-3′

The recombinant vectors with the DNA fragment were transformed into E. coli DH5α competent cells. After PCR and sequencing, the positive plasmids were selected.

Site-directed mutagenesis to generate the active-site mutant *Op5*Man5 variant E156Q/E281Q was performed using the following forward and reverse primers:

E156Q _fwd (5′- GATCTGGGCAACCAGCTGGATGCGCTC −3′)

E156Q _rev (5′- GAGCGCATCCAGCTGGTTGCCCAGATC −3′)

E281Q_fwd (5′- CCGGTCCTGTTACAGCAGTTTGGCACCATTCTC −3′)

E281Q_rev (5′- GAGAATGGTGCCAAACTGCTGTAACAGGACCGG −3′)

The wild-type gene sequence was used as template for generating the single mutants E156Q and E281Q whereas E156Q sequence was used as a template for generating the double mutant E156Q/E281Q. Mutagenesis was performed using the QuikChange II site-directed mutagenesis kit (Agilent Technologies) as described by the manufacturer. The mutated gene sequences were confirmed by DNA sequencing, and the plasmids with confirmed mutations were transformed into *E. coli* BL21 (DE3)-T1R for protein production.

### Production and purification of *Op5*Man5 and active-site mutants

The insert sequence was verified, and the plasmid transformed into the BL21 (DE3) T1R strain. The cells were cultured overnight in (50 ml) Terrific Broth (TB) medium with kanamycin (50 μg ml^−1^) and 0.4% glycerol (v/v) at 30 °C, 175 r.p.m. A small volume (1%) overnight culture was used to inoculate 2 L of TB medium supplemented with 0.8% glycerol (v/v) and kanamycin (50 μg ml^−1^). The culture was incubated at 37 °C, 180 r.p.m. until OD_600_ 2, after which the temperature was lowered to 18 °C. Protein expression was induced at OD_600_ 3 with the addition of IPTG to a final concentration of 0.5 mM, and the culture was incubated overnight at 18 °C. The cells were harvested, and the pellet re-suspended in lysis buffer (100 mM HEPES, 500 mM NaCl, 10% glycerol, 10 mM TCEP 25 mM, pH 8.0) with one tablet of cOmplete^TM^ protease inhibitor cocktail (Roche). The suspended pellet was homogenized using an AVESTIN Emulsiflex-C3 system, and further disrupted by pulsed sonication (4 s/4s 3 min, 80% amplitude).

The lysate was centrifuged (20 min at 49000 × *g*) using a Beckman Ti 45 rotor, and the resulting supernatant filtered through a 0.45-μm filter. The resulting supernatant was filtered through a 0.45-μM filter. The resulting supernatant was loaded onto a Ni–NTA HisTrap HP column (5 ml, GE Healthcare) using an ÄKTA start system (GE Healthcare, Uppsala, Sweden). The columns were washed with 20 mM HEPES buffer (pH 7.5), 500 mM NaCl, 50 mM imidazole, 10% (v/v) glycerol, 0.5 mM TCEP. Bound protein was eluted with buffer containing 500 mM imidazole. Protein samples were concentrated using Vivaspin20 centrifugal concentrators (polyethersulfone filter, molecular weight cut-off, MWCO, 30 kDa), and loaded onto a HiLoad 16/60 Superdex 200 prep grade column (GE Healthcare Life Sciences) equilibrated with 50 HEPES buffer (pH 7.5) 300 mM NaCl, 10% (v/v) glycerol and 2 mM TCEP. The fractions corresponding to the middle of the eluted peak were pooled and checked for purity by SDS-PAGE and liquid chromatography-tandem mass spectrometry (LC-MS/MS) analysis. The fractions containing *Op5*Man5 were recovered and pooled, and concentrated using a Vivaspin20 concentrator (MWCO 30 kDa) to a final concentration of 5–10 mg ml^−1^. The *Op5*Man5 mutant (E194Q/E294Q) was produced as described for the wild type.

### Substrate screening

Different aryl glycosides were screened to identify suitable substrates. The reaction mixture contained 0.2 ml of 1 mM pNP substrate in 50 mM sodium phosphate (pH 6.0) and 5 μl of enzyme (2 mg ml^−1^). After incubation at 40 °C for 10 min, the reaction was stopped by adding 20 μl 2 M Na_2_CO_3_. The released p-nitrophenol product was measured using a spectrophotometer at 410 nm. Enzyme and substrate free controls were also incubated and measured under the same conditions. All enzyme assays were performed in triplicate and one unit of enzyme activity (U) was defined as the amount of enzyme that produces one μmol of pNP per minute.

Substrate screening of oligosaccharides and polysaccharides was performed using the GOD-POD assay. Unless otherwise indicated, assay mixtures contained substrate and appropriately diluted enzyme in 50 mM sodium phosphate buffer (pH 6.0) at 40 °C. Briefly, appropriate concentration of *Op5*Man5 was mixed with 500 μl of polymeric substrate (5 mg ml^−1^) or 100 μl oligosaccharides (5 mM) and the reaction was terminated by heating the sample at 100 °C for 5 min. One unit of enzyme activity was defined as the amount of protein that released 1 μmol min^−1^ of glucose.

### Effect of pH and temperature on enzyme activity

The effects of pH on *Op5*Man5 activity were tested using 50 mM sodium acetate buffer (pH 4.0–5.5), sodium phosphate buffer (pH 6.0–7.5), and tris buffer (pH 8.5–9.0) at 40 °C using *p*NP-*β*Man in sodium phosphate buffer (pH 6.0). The effect of temperature (*T*) on activity was investigated in temperature range 20 °C to 70 °C. The residual activity was expressed as a percentage of the highest activity. The stability of activity as a function of *T* was determined by incubating *Op5*Man5 (0.02 mg ml^−1^) at *T* (°C) = 40, 50, 60, and 70. Aliquots were taken at different time points (samples were taken at the time points: 0, 5, 10, 20, 30, 40, 60, 80 min; and 2, 4, 6, 12, 18, 24 h) and assayed as described above using *p*NP-*β*Man as substrate. Data were collected in triplicate, and the half-time of irreversible thermal inactivation (*a_t_*) was calculated by non-linear regression using the exponential decay equation in SigmaPlot (Systat Software, San Jose, CA): $a_{t}={a_{0}}^{\left(-t*ln\frac{2}{T_{1/2}}\right)}$

### Effect of metal ions

The effect of metal ions (NiCl_2,_ FeCl_3,_ CaCl_2_, CuCl_2_, MgCl_2_, ZnCl_2,_ CrSO_4_ and MnCl_2_) on *Op5*Man5 activity was investigated with *p*NP-*β*Man as substrate using the standard assay described above. Before starting the reaction, *Op5*Man5 (2 mg ml^−1^) was pre-incubated in 50 mM sodium phosphate (pH 6.0) in the presence of either 2 or 5 mM metal ion for 5 min at 40 °C. The reaction was started by adding 0.2 ml of 1 mM *p*NP-*β*Man in 50 mM sodium phosphate (pH 6.0). The remaining activity was measured after incubation for 10 min at 50 °C. The residual activity was determined with reference to the standard reaction without added metal ions set to 100%. The negative control contained *Op5*Man5 that had been treated with 1 mM EDTA. All reactions were performed in triplicate with the standard deviation errors calculated.

### Determination of kinetic parameters for *p*NP-*β*Man and MOSs

The kinetic parameters, Michaelis-Menten constant (K_m_), maximum velocity (V_max_), and turnover number (k_cat_), were determined using *p*NP-*β*Man as substrate. Reaction mixtures (200 μl) contained 0.5–3.0 mM (at the points 0.025, 0.5, 1.0, 1.5, 2.0, 2.5 and 3.0 mM) *p*NP-*β*Man 50 mM sodium phosphate buffer (pH 6.0), to which 5 μl *Op5*Man5 (2 mg ml^−1^) was added to start the reaction (40 °C for 10 min). The reaction was stopped by adding 20 μl 2 M Na_2_CO_3_. All enzyme assays were performed in triplicate. Values of K_m_, V_max_ and k_cat_ were calculated by nonlinear regression using Michaelis-Menten equation fit using the GraphPad Prism 5 kinetic analysis package (GraphPad Software, San Diego, CA).

For the MOSs (M4-M6), initial velocity of each reaction was measured within 12 h using high-performance anion exchange chromatography (HPAEC) and the *k*_cat_/*K*_m_ values were derived from a linear fit *v* = [E_0_][S_0_](*k*_cat_/*K*_m_). HPAEC (Dionex ICS5000, Sunnyvale, CA, USA) equipped with a Carbo-Pac PA200 column (3 μm × 250 mm) and a pulsed amperometric detector (PAD) was used to analyze hydrolysis products. The hydrolysates were diluted in deionized water (1:100 v/v), and 10 μl of sample was injected in a PA200 column with an isocratic flow of 0.5 ml⋅min^−1^ of 100 mM NaOH at 30 °C for 35 min. Peak assignment was performed by comparison of the retention time with a series of standard MOSs. Calibration was performed with standard solutions at concentrations in the range 0.01 to 0.2 mM. The peak area was used for quantification.

### TLC analysis of products from MOS hydrolysis

Hydrolysis products obtained from MOSs (M2, M3, M4, M5, and M6) were analyzed by TLC. *Op5*Man5 (1 μl of 2 mg ml^−1^) was added to buffered MOS solutions (50 μl of 10 M2-M6), and the reaction allowed to proceed for 72 h at 40 °C. Samples were withdrawn at different time points (0, 12, 24, 48, 72 h), and the reactions terminated by heating the samples in a 100 °C water bath for 10 min. After centrifugation (10,000 × *g*, 10 min), the samples were spotted on Silica gel 60 F_254_ plates (Merck Millipore) and air-dried. TLC was performed using a mobile solvent consisting of butanol:propanol:ethanol:water (2:3:3:2, v/v), and the silica plates were stained with thymol and heated for 10 min at 120 °C. The control samples were treated identically, with the exception of using heat-inactivated enzyme (100 °C, 10 min).

### Transglycosylation of mannobiose and alcohols by Op5Man5

To demonstrate whether alcohols can act as acceptors for glycosyl moieties, various alcohols (500 mM) were used as acceptors and mixed with mannobiose (5 mM) followed by incubation with 5 μl (2 mg ml^−1^) *Op5*Man5 in 50 mM phosphate buffer (pH 6.0) at 40 °C for 24 h. The transfer products were analyzed using TLC as described above.

### Crystallization and X-ray intensity data collection

Crystals were obtained at room temperature from a solution containing 20 mg ml^−1^ *Op5*Man5 in 0.1 M phosphorus citrate (pH 4.2), 30% (v/v) ethanol, and 5% (w/v) polyethylene glycol 1000 using the vapor diffusion method and sitting drops. Crystals were subjected to soaking with 1 mM UO_2_(NO_3_)_2_ with the aim to obtain heavy-atom phases. Synchrotron data were recorded on crystals vitrified in liquid nitrogen (100 K) at Diamond Light Source beamline *I*03 to 2.2 Å resolution, and scaled using the *XDS* software package (Kabsch, 2010) (Table S2). The crystals belong to space group *P*2_1_2_1_2_1_ with the cell dimensions *a* = 102.14 Å, *b* = 162.45, *c* = 168.18 Å, *α* = *β* = *γ* = 90° with three molecules (one homotrimer) in the asymmetric unit.

### Grid preparation, cryo-EM and image processing

Aliquots of 3 µl of purified *Op5*Man5 (0.12 mg ml^−1^) were applied to glow-discharged (60 s, 20 mA using Quorum GloQube), 300-mesh (copper) C-Flat (R2/2) carbon grids (EMS, USA). Using a FEI Vitrobot Mark IV (Thermo Fischer Scientific) operating at 4 °C and 100% humidity, grids were blotted for 3 s before being flash-frozen into liquid ethane. Microscopy data was collected using a Talos Arctica electron microscope (Thermo Fischer Scientific) operated at 200 kV equipped with a Falcon III direct electron detector (Thermo Fischer Scientific). A total of 338 movies were acquired automatically with *EPU* (Thermo Fischer Scientific), using single*‐*electron counting mode at a nominal magnification of 150,000 × (0.99 Å pixel^−1^), a flux of 0.56 e Å^2–1^ s^−1^ and a total dose of 60 e Å^2–1^ over a total of 24 frames with defoci ranging from −0.5 μm to −3 μm.

All of the following data processing steps were performed in *cryoSPARC* version 2.12 (Punjani et al., 2017). Frames were aligned, averaged and dose*‐*weighted with Patch motion (Rubinstein and Brubaker, 2015) and the contrast transfer functions for each micrograph was estimated with Patch CTF. 67 micrographs with a CTF fit quality above 5 µm were excluded. Template-based particle picking yielded 179,684 particles of which 22,453 spurious particles were removed by 2D classification, yielding a dataset of ∼ 157231 particles. Through *ab*-*initio* reconstruction and heterogeneous refinement, a class with 85,735 particles could be identified and was subsequently refined via homogeneous and non-uniform refinement to 3.7 Å, for which per particle defoci were optimised and higher order aberrations corrected (Zivanov et al., 2019). The reported resolution is based on the Fourier shell correlation between two independently refined half maps (Scheres and Chen, 2012) using a 0.143 criterion (Rosenthal and Henderson, 2003), and the FSC*‐*curve was corrected for the convolution effects of a soft mask using high*‐*resolution noise substitution (Chen et al., 2013). The density map was locally filtered and sharpened by applying a negative B*‐*factor (-220 Å^2^) that was estimated using automated procedures (Rosenthal and Henderson, 2003).

### Crystal-structure determination and model refinement

A partial model was produced from the 3.7-Å cryo-EM potential map using the model-building option (*Map to model*, 3 building cycles) in the *Phenix* suite (Adams et al., 2010). The partial cryo-EM model was used for molecular replacement against the 2.2-Å X-ray amplitudes with *Phenix Phaser* (Adams et al., 2010). A clear solution was found for the three monomers of the homotrimer in the asymmetric unit of the *P*2_1_2_1_2_1_ cell (313.8, TFZ 20.1). The MR phases were further subjected to improvement by density modification and three-fold averaging using *Phenix Resolve*. The improved phases were of high quality and allowed building of a nearly complete model (1651 residues of 1755) using automatic building with *Phenix AutoBuild*, which generated a model with 1626 residues and *R* and *R*_free_ values of 0.205 and 0.243, respectively. The *Op5*Man5 model was refined with *phenix.refine* and adjusted iteratively by hand with *Coot* (Emsley and Cowtan, 2004) with guidance from *σ*_A_-weighted 2*F*_o_-*F*_c_ electron-density map at 2.2-Å resolution. The final model contained 1637 residues and 1056 solvent molecules (*R*/*R*_free_ 0.201/0.237). A comparison between the initial 3.7 Å cryo-EM map and the 2.2 Å 2*F*_o_-*F*_c_ electron-density map is shown in Figure S11. The atomic coordinates and structure factor amplitudes have been deposited in the Protein Data Bank (www.rcsb.org) with the accession code 7BOB.

### Analysis of oligomeric state using SEC and crosslinking

The oligomeric state of *Op5*Man5 was investigated using size-exclusion chromatography (SEC) on a Superdex 200 10/300 GL column equilibrated with 20 mM buffer supplemented with 300 mM NaCl and different pH values (sodium acetate pH 5.5; MES pH 6.0 and 6.5; HEPES pH 7.5; Tris-HCl pH 8.0 and 8.5) at a flow rate of 1 ml⋅min^−1^ at 4 °C. For estimation of the *Op5*Man5 molecular weight, standard proteins included ferritin (440 kDa), Aldolase (158 kDa), conalbumin (75 kDa), and ribonuclease A (13.7 kDa). Glutaraldehyde (Sigma-Aldrich) was added to the 200 μl of protein sample (0.2 mg ml^−1^) at final concentration of 0.4% w/v. Samples were incubated at 37 °C for 0 to 60 min, and samples (15 μl) were taken at different time points: 0, 5, 15, 30 and 60 min. The reactions were stopped by adding 1 M Tris-HCl buffer pH 8.0 (2.2 μl), and analyzed using SDS-PAGE (15%).

### Sequence analyses

Analysis of signal peptides was performed using SignaIP-5.0 (Almagro Armenteros et al., 2019). Structure-based sequence alignment was performed manually by interactively analyzing the overlaid protein structures using *Coot* (Emsley and Cowtan, 2004). The aligned sequences were visualized using ESpript 3 (espript.ibcp.fr (Robert and Gouet, 2014). For generating the phylogenetic tree, BLASTP was first used to identify and align the amino-acid sequences, after which maximum likelihood phylogenetic analysis was performed using PhyML (www.phylogeny.fr (Dereeper et al., 2008). The phylogenetic tree was generated using Interactive Tree Of Life (iTOL) v 5.7 (Letunic and Bork, 2019).

## CRediT authorship contribution statement

**Dayanand C. Kalyani:** Methodology, Validation, Formal analysis, Investigation, Writing - original draft, Writing - review & editing, Visualization. **Tom Reichenbach:** Methodology, Validation, Formal analysis, Investigation, Writing - original draft. **Markus M. Keskitalo:** Validation, Formal analysis, Investigation, Writing - original draft. **Julian Conrad:** Methodology, Validation, Formal analysis, Investigation, Resources, Writing - original draft, Supervision. **Henrik Aspeborg:** Conceptualization, Methodology, Validation, Formal analysis, Investigation, Resources, Writing - original draft, Supervision, Project administration, Funding acquisition. **Christina Divne:** Conceptualization, Methodology, Validation, Formal analysis, Investigation, Resources, Writing - original draft, Visualization, Supervision, Project administration, Funding acquisition.

## Declaration of Competing Interest

The authors declare that they have no known competing financial interests or personal relationships that could have appeared to influence the work reported in this paper.

## References

[b0005] Govorushko S. (2019). Economic and ecological importance of termites: A global review. Entomolog. Sci..

[b0010] Peterson B.F., Scharf M.E. (2016). Lower termite associations with microbes: synergy, protection, and interplay. Front. Microbiol..

[b0015] Su N.Y., Scheffrahn R.H. (1990). Economically important termites in the United States and their control. Sociobiology.

[b0020] Karl Z.J., Scharf M.E. (2015). Effects of five diverse lignocellulosic diets on digestive enzyme biochemistry in the termite *Reticulitermes flavipes*. Arch. Insect Biochem. Physiol..

[b0025] Rajarapu S.P., Shreve J.T., Chide K.P., Thimmapuram J., Scharf M.E. (2015). Metatranscriptomic profiles of Eastern subterranean termites, *Reticulitermes flavipes* (Kollar) fed on second generation feedstocks. BMC Genomics.

[b0030] Rajarapu S.P., Scharf M.E. (2017). Saccharification of agricultural lignocellulose feedstocks and protein-level responses by a termite gut-microbe bioreactor. Front. Energy Res..

[b0035] Boucias D.G., Cai Y., Sun Y., Lietze V.-U., Sen R., Raychoudhury R., Scharf M.E. (2013). The hindgut lumen prokaryotic microbiota of the termite *Reticulitermes flavipes* and its responses to dietary lignocellulose composition. Mol. Ecol..

[b0040] Kotak M., Isanapong J., Goodwin L., Bruce D., Chen A., Han C.S., Huntemann M., Ivanova N., Land M.L., Nolan M., Pati A., Woyke T., Rodrigues J.L. (2015). Complete genome sequence of the *Opitutaceae* bacterium strain TAV5, a potential facultative methylotroph of the wood-feeding termite *Reticulitermes flavipes*. Genome Announce.

[b0045] Nixon S.L., Daly R.A., Borton M.A., Solden L.M., Welch S.A., Cole D.R., Mouser P.J., Wilkins M.J., Wrighton K.C., Suen G. (2019). Genome-resolved metagenomics extends the environmental distribution of the Verrucomicrobia phylum to the deep terrestrial subsurface..

[b0050] Martinez-Garcia M., Brazel D.M., Swan B.K., Arnosti C., Chain P.S.G., Reitenga K.G., Xie G., Poulton N.J., Gomez M.L., Masland D.E.D., Thompson B., Bellows W.K., Ziervogel K., Lo C.-C., Ahmed S., Gleasner C.D., Detter C.J., Stepanauskas R., Ravel J. (2012). Capturing single cell genomes of active polysaccharide degraders: An unexpected contribution of *Verrucomicrobia*. PLoS One.

[b0055] Herlemann D.P.R., Lundin D., Labrenz M., Jürgens K., Zheng Z., Aspeborg H., Andersson A.F., Azam F., Simon M. (2013). Metagenomic de novo assembly of an aquatic representative of the verrucomicrobial class *Spartobacteria*. mBio.

[b0060] Cardman Z., Arnosti C., Durbin A., Ziervogel K., Cox C., Steen A.D., Teske A., Spormann A.M. (2014). Verrucomicrobia are candidates for polysaccharide-degrading bacterioplankton in an arctic fjord of Svalbard. Appl. Environ. Microbiol..

[b0065] Fan, L. T., Lee, Y.-H. & Gharpuray, M. M. (1982). The nature of lignocellulosics and their pretreatments for enzymatic hydrolysis. Advances in Biochemical Engineering, vol 23. Springer, Berlin, Heidelberg. https://doi.org/10.1007/3540116982_4.

[b0070] Malgas S., van Dyk J.S., Pletschke B.I. (2015). A review of the enzymatic hydrolysis of mannans and synergistic interactions between β-mannanase, β-mannosidase and α-galactosidase. World J. Microbiol. Biotechnol..

[b0075] Eneyskaya E.V., Sundqvist G., Golubev A.M., Ibatullin F.M., Ivanen D.R., Shabalin K.A., Brumer H., Kulminskaya A.A. (2009). Transglycosylating and hydrolytic activities of the beta-mannosidase from *Trichoderma reesei*. Biochimie.

[b0080] Zhang M., Jiang Z., Li L., Katrolia P. (2009). Biochemical characterization of a recombinant thermostable β-mannosidase from *Thermotoga maritima* with transglycosidase activity. J. Mol. Catal..

[b0085] Zhou P., Liu Y., Yan Q., Chen Z., Qin Z., Jiang Z. (2014). Structural insights into the substrate specificity and transglycosylation activity of a fungal glycoside hydrolase family 5 β-mannosidase 2014. Acta Crystallogr. D Biol. Crystallogr..

[b0090] Lombard V., Golaconda Ramulu H., Drula E., Coutinho P.M., Henrissat B. (2014). The carbohydrate-active enzymes database (CAZy) in 2013. Nucleic Acids Res.

[b0095] Shallom D., Shoham Y. (2003). Microbial hemicellulases. Curr. Opin. Microbiol..

[b0100] Dias F.M.V., Vincent F., Pell G., Prates J.A.M., Centeno M.S.J., Tailford L.E., Ferreira L.M.A., Fontes C.M.G.A., Davies G.J., Gilbert H.J. (2004). Insights into the molecular determinants of substrate specificity in glycoside hydrolase family 5 revealed by the crystal structure and kinetics of *Cellvibrio mixtus* mannosidase 5A. J. Biol. Chem..

[b0105] Xie J., He Z., Wang Z., Wang B., Pan L.i. (2019). Efficient expression of a novel thermophilic fungal β-mannosidase from *Lichtheimia ramosa* with broad-range pH stability and its synergistic hydrolysis of locust bean gum. J. Biosci. Bioeng..

[b0110] Gytz H., Liang J., Liang Y., Gorelik A., Illes K., Nagar B. (2019). The structure of mammalian β*-*mannosidase provides insight into β*-*mannosidosis and nystagmus. FEBS J..

[b0115] Hwa K.Y., Subramani B., Shen S.T., Lee Y.M. (2015). Exchange of active site residues alters substrate specificity in extremely thermostable β-glycosidase from *Thermococcus kodakarensis* KOD1. Enz. Microb. Technol..

[b0120] Shi H., Huang Y., Zhang Y.u., Li W., Li X., Wang F. (2013). High-level expression of a novel thermostable and mannose-tolerant β-mannosidase from *Thermotoga thermarum* DSM 5069 in *Escherichia coli*. BMC Biotechnol..

[b0125] Tailford L.E., Money V.A., Smith N.L., Dumon C., Davies G.J., Gilbert H.J. (2007). Mannose foraging by *Bacteroides thetaiotaomicron*: structure and specificity of the β-mannosidase, BtMan2A. J. Biol. Chem..

[b0130] Stoll D., Stålbrand H., Warren R.A.J. (1999). Mannan-degrading enzymes from *Cellulomonas fimi*. Appl. Environ. Microbiol..

[b0135] Guillotin L., Richet N., Lafite P., Daniellou R. (2017). Is the acid/base catalytic residue mutation in β-D-mannosidase DtMan from *Dictyoglomus thermophilum* sufficient enough to provide thioglycoligase activity?. Biochimie.

[b0140] Shi P., Yao G., Cao Y., Yang P., Yuan T., Huang H., Bai Y., Yao B. (2011). Cloning and characterization of a new β-mannosidase from Streptomyces sp. S27. Enz. Microb. Technol..

[b0145] Prendecka M., Buczynska A., Rogalski J. (2007). Purification and characterization of β-mannosidases from white rot fungus Phlebia radiata. Pol. J. Microbiol..

[b0150] Li Y., Liu Y., Yan Q., Yang S., Jiang Z. (2015). Characterization of a novel glycoside hydrolase family 5 β-mannosidase from *Absidia corymbifera* with high transglycosylation activity. J. Mol. Catal. B.

[b0155] Reichenbach T., Kalyani D., Gandini R., Svartström O., Aspeborg H., Divne C., Soares C.M. (2018). Structural and biochemical characterization of the *Cutibacterium acnes* exo-β-1,4-mannosidase that targets the *N*-glycan core of host glycoproteins. PLoS One.

[b0160] Zahura U.A., Rahman M.M., Inoue A., Ojima T. (2012). Characterization of a β-D-mannosidase from a marine gastropod, *Aplysia kurodai*. *Comp. Biochem. Physiol. B*. Biochem. Mol. Biol..

[b0165] Xu Z., Escamilla-Treviño L., Zeng L., Lalgondar M., Bevan D., Winkel B., Mohamed A., Cheng C.-L., Shih M.-C., Poulton J., Esen A. (2004). Functional genomic analysis of Arabidopsis thaliana glycoside hydrolase family 1. Plant Mol. Biol..

[b0170] McCleary B.V. (1983). β-D-mannosidase from *Helix pomatia*. Carbohydr. Res..

[b0175] Akino T., Nakamura N., Horikoshi K. (1988). Characterization of β-mannosidase of an alkalophilic *Bacillus* sp. Agric. Biol. Chem..

[b0180] Gübitz G.M., Hayn M., Sommerauer M., Steiner W. (1996). Mannan-degrading enzymes from Sclerotium rolfsii: Characterisation and synergism of two endo β-mannanases and a β-mannosidase. Bioresour. Technol..

[b0185] Andreotti G., Giordano A., Tramice A., Mollo E., Trincone A. (2005). Purification and characterization of a β-D-mannosidase from the marine anaspidean *Aplysia fasciata*. J. Biotechnol..

[b0190] Lundemo P., Adlercreutz P., Karlsson E.N. (2013). Improved transferase/hydrolase ratio through rational design of a family 1 β-glucosidase from *Thermotoga neapolitana*. Appl. Environ. Microbiol..

[b0195] Morrill J., Månberger A., Rosengren A., Naidjonoka P., von Freiesleben P., Krogh K.B.R.M., Bergquist K.-E., Nylander T., Karlsson E.N., Adlercreutz P., Stålbrand H. (2018). β-Mannanase-catalyzed synthesis of alkyl mannooligosides. Appl. Microbiol. Biotechnol..

[b0200] Kurakake M., Komaki T. (2001). Production of β-mannanase and β-mannosidase from *Aspergillus awamori* K4 and their properties. Curr. Microbiol..

[b0205] Rosengren A., Reddy S.K., Sjöberg J.S., Aurelius O., Logan D.T., Kolenová K., Stålbrand H. (2014). An *Aspergillus nidulans* β-mannanase with high transglycosylation capacity revealed through comparative studies within glycosidase family 5. Appl. Microbiol. Biotechnol..

[b0210] Kelley L.A., Mezulis S., Yates C.M., Wass M.N., Sternberg M.J.E. (2015). The Phyre2 web portal for protein modeling, prediction and analysis. Nat. Protoc..

[b0215] Hilge M., Gloor S.M., Rypniewski W., Sauer O., Heightman T.D., Zimmermann W., Winterhalter K., Piontek K. (1998). High-resolution native and complex structures of thermostable β-mannanase from *Thermomonospora fusca* – substrate specificity in glycosyl hydrolase family 5. Structure.

[b0220] Fan Y., Yi J., Hua X., Feng Y., Yang R., Zhang Y. (2016). Structure analysis of a glycosides hydrolase family 42 cold-adapted β-galactosidase from *Rahnella* sp. R3. RSC Advances.

[b0225] Viborg A.H., Katayama T., Arakawa T., Abou Hachem M., Lo Leggio L., Kitaoka M., Svensson B., Fushinobu S. (2017). Discovery of α-L-arabinopyranosidases from human gut microbiome expands the diversity within glycoside hydrolase family 42. J. Biol. Chem..

[b0230] Armstrong Z., Davies G.J. (2020). Structure and function of Bs164 β-mannosidase from *Bacteroides salyersiae* the founding member of glycoside hydrolase family GH164. J. Biol. Chem..

[b0235] Cordeiro R.L., Pirolla R.A.S., Persinoti G.F., Gozzo F.C., de Giuseppe P.O., Murakami M.T. (2019). N-glycan utilization by *Bifidobacterium* gut symbionts involves a specialist β-mannosidase. J. Mol. Biol..

[b0240] Kabsch W. (2010). XDS. Acta Crystallogr. D Biol. Crystallogr..

[b0245] Punjani A., Rubinstein J.L., Fleet D.J., Brubaker M.A. (2017). cryoSPARC: algorithms for rapid unsupervised cryo-EM structure determination. Nat. Methods.

[b0250] Rubinstein J.L., Brubaker M.A. (2015). Alignment of cryo-EM movies of individual particles by optimization of image translations. J. Struct. Biol..

[b0255] Zivanov J., Nakane T., Scheres S.H.W. (2019). Estimation of High-Order Aberrations and Anisotropic Magnification from Cryo-EM Datasets in RELION-3.1. IUCrJ.

[b0260] Scheres S.H.W., Chen S. (2012). Prevention of overfitting in cryo-EM structure determination. Nat. Methods.

[b0265] Rosenthal P.B., Henderson R. (2003). Optimal determination of particle orientation, absolute hand, and contrast loss in single-particle electron cryomicroscopy. J. Mol. Biol..

[b0270] Chen S., McMullan G., Faruqi A.R., Murshudov G.N., Short J.M., Scheres S.H., Henderson R. (2013). High-resolution noise substitution to measure overfitting and validate resolution in 3D structure determination by single particle electron cryomicroscopy. Ultramicroscopy.

[b0275] Adams P.D., Afonine P.V., Bunkóczi G., Chen V.B., Davis I.W., Echols N., Headd J.J., Hung L.-W., Kapral G.J., Grosse-Kunstleve R.W., McCoy A.J., Moriarty N.W., Oeffner R., Read R.J., Richardson D.C., Richardson J.S., Terwilliger T.C., Zwart P.H. (2010). PHENIX: a comprehensive Python-based system for macromolecular structure solution. Acta Crystallogr. D Biol. Crystallogr..

[b0280] Emsley P., Cowtan K. (2004). Coot: model-building tools for molecular graphics. Acta Crystallogr. D Biol. Crystallogr..

[b0285] Almagro Armenteros J.J., Tsirigos K.D., Sønderby C.K., Petersen T.N., Winther O., Brunak S., von Heijne G., Nielsen H. (2019). SignalP 5.0 improves signal peptide predictions using deep neural networks. Nat. Biotechnol..

[b0290] Robert X., Gouet P. (2014). Deciphering key features in protein structures with the new ENDscript server. Nucleic Acids Res..

[b0295] Dereeper A., Guignon V., Blanc G., Audic S., Buffet S., Chevenet F., Dufayard J.-F., Guindon S., Lefort V., Lescot M., Claverie J.-M., Gascuel O. (2008). Phylogeny.fr: robust phylogenetic analysis for the non-specialist. Nucleic Acids Res..

[b0300] Letunic I., Bork P. (2019). Interactive Tree Of Life (iTOL) v4: recent updates and new developments. Nucleic Acids Res..

