# Supplementary material for: Crystal structure of a homotrimeric verrucomicrobial exo-β-1,4-mannosidase active in the hindgut of the wood-feeding termite Reticulitermes flavipes
Source: J Struct Biol X. 2021 May 24;5:100048. doi: 10.1016/j.yjsbx.2021.100048 (PMC8233224; doi:10.1016/j.yjsbx.2021.100048)
Supplement: Supplementary data 1 [file mmc1.pdf]

Appendix A. Supplementary data

Crystal structure of a homotrimeric verrucomicrobial exo-β-1,4-mannosidase active in the hindgut of the wood-feeding termite *Reticulitermes flavipes*

Dayanand C. Kalyani[1#], Tom Reichenbach[1#], Markus M. Keskitalo[1], Julian Conrad[2,3], Henrik Aspeborg[1], Christina Divne[1]

1 - School of Engineering Sciences in Chemistry, Biotechnology, and Health *(CBH), KTH Royal Institute of Technology, Roslagstullsbacken 21, SE-11421 Stockholm, Sweden.*

2 - Department of Biochemistry and Biophysics*, Stockholm University, Svante Arrhenius väg 16C, SE-10691 Stockholm, Sweden.*

3 - Science for Life Laboratory*, Stockholm University, Tomtebodavägen 23, SE-17165 Solna, Sweden.*

[#] These authors contributed equally to this work.

Correspondence to Christina Divne: *divne@kth.se*

Figure S1

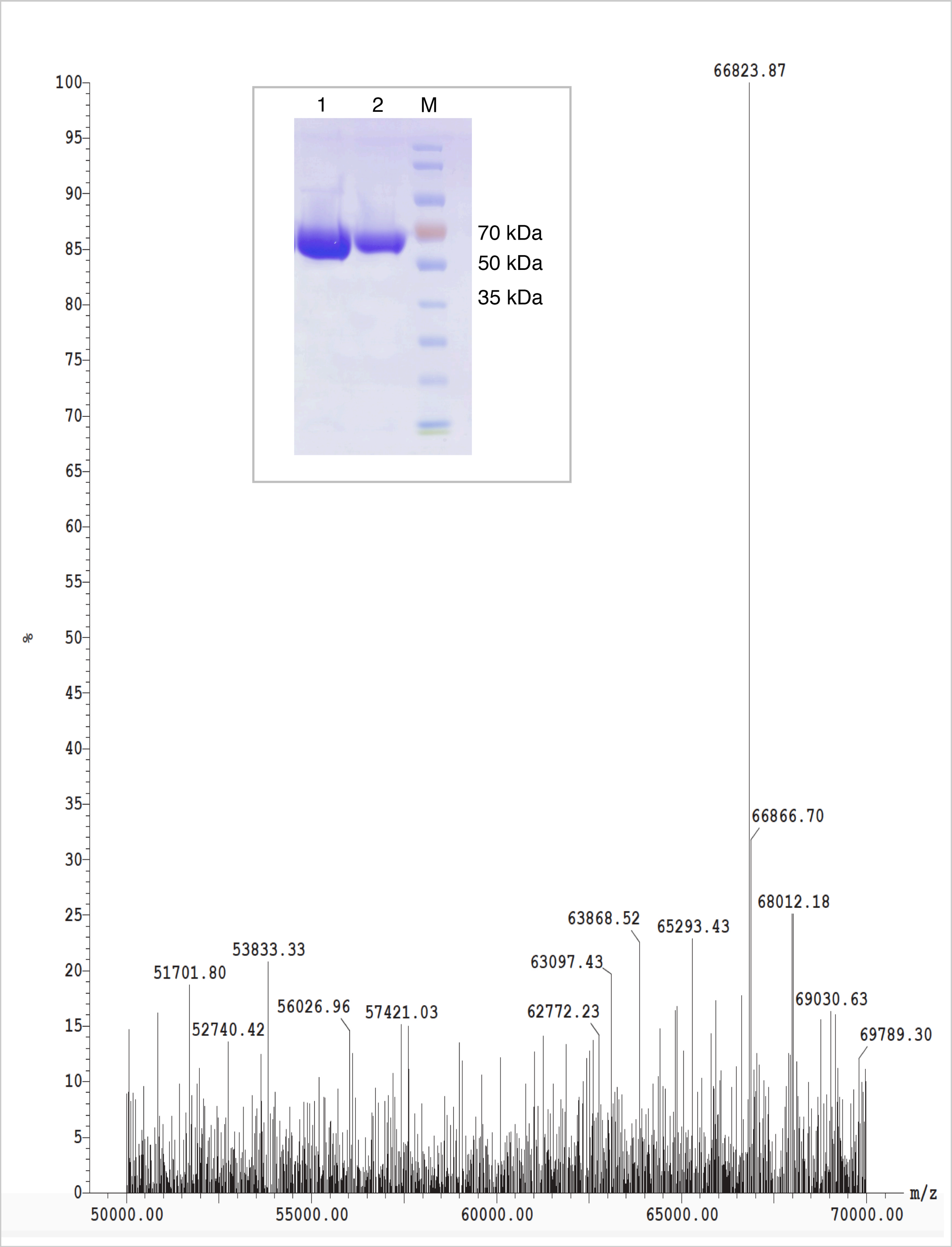

Figure S1. Molecular mass of *Op5*Man5. Liquid chromatography-tandem mass spectrometry (LC-MS/MS) of recombinant *Op5*Man5 gives a molecular mass of 66,824 g/mol. The inset panel shows an SDS-PAGE gel of purified *Op5*Man5 with different amounts of protein loaded in the two lanes. M, molecular weight reference proteins.

Figure S2

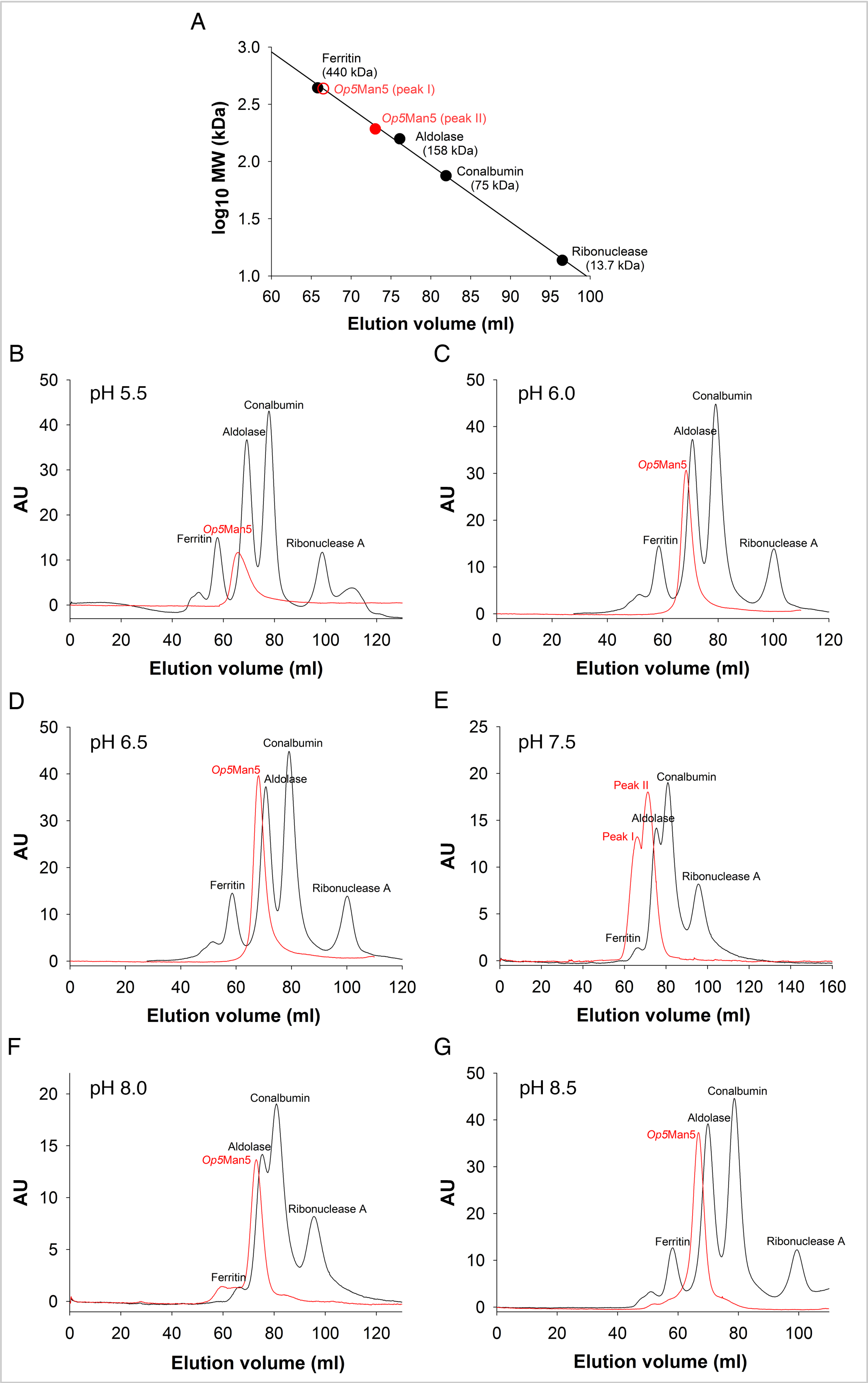
A
log10 MW (kDa)
Elution volume (ml)
Ferritin
(440 kDa)
Op5Man5 (peak I)
Op5Man5 (peak II)
Aldolase
(158 kDa)
Conalbumin
(75 kDa)
Ribonuclease
(13.7 kDa)
B
pH 5.5
C
pH 6.0
D
pH 6.5
E
pH 7.5
Peak II
Peak I
F
pH 8.0
G
pH 8.5
AU
Ferritin
Aldolase
Conalbumin
Ribonuclease A
Op5Man5

Figure S2. SEC analysis of *Op5*Man5. (A) Standard curve at pH 7.5 (R^2 =0.9954) based on the reference proteins (black filled circles), and the extrapolated molecular weights of the *Op5*Man5 hexamer (peak I; ~434 kDa; red open circle) and trimer (peak II; ~214 kDa; red filled circle). SEC profiles of *Op5*Man5 at different pH values (red curve) overlaid with the profiles of the reference proteins: (B) pH 5.5; (C) pH 6.0; (D) pH 6.5; (E) pH 7.5; (F) pH 8.0; and (G) pH 8.5. The SEC profile in (E) shows two distinct species: peak I (hexamer) and peak II (trimer).

Figure S3

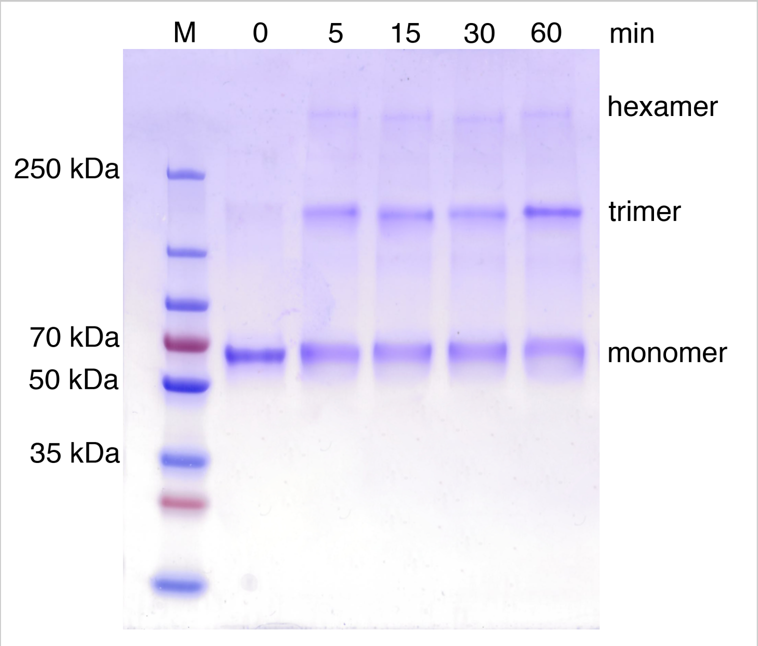

Figure S3. Chemical cross-linking of *Op5*Man5. Results of the crosslinking reaction as a function of time as represented by a Coomassie-stained SDS-PAGE. At t = 0, only the monomer band is present.

Figure S4

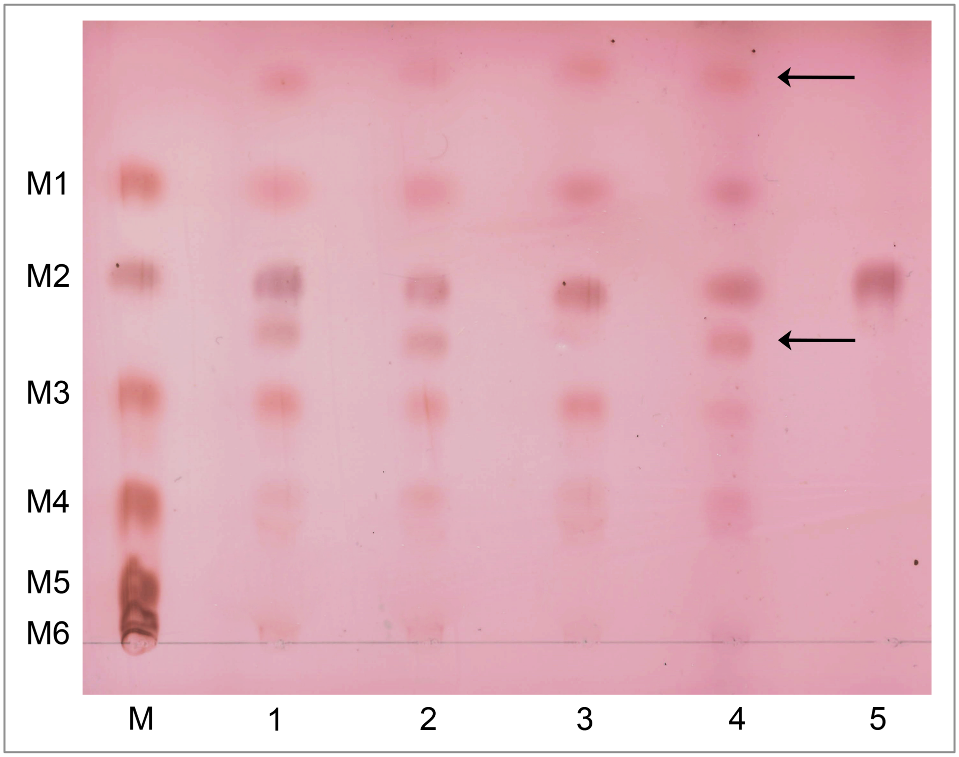

Figure S4. Transglycosylation products. TLC analysis of products from incubating M2 with methanol (lane 1), ethanol (lane 2), 1-propanol (lane 3), and hexanol (lane 4). Reference mannooligosaccharides: M1-M6 (lane M), and M2 (lane 5). Alkyl β-mannosides are indicated by arrows.

Figure S5

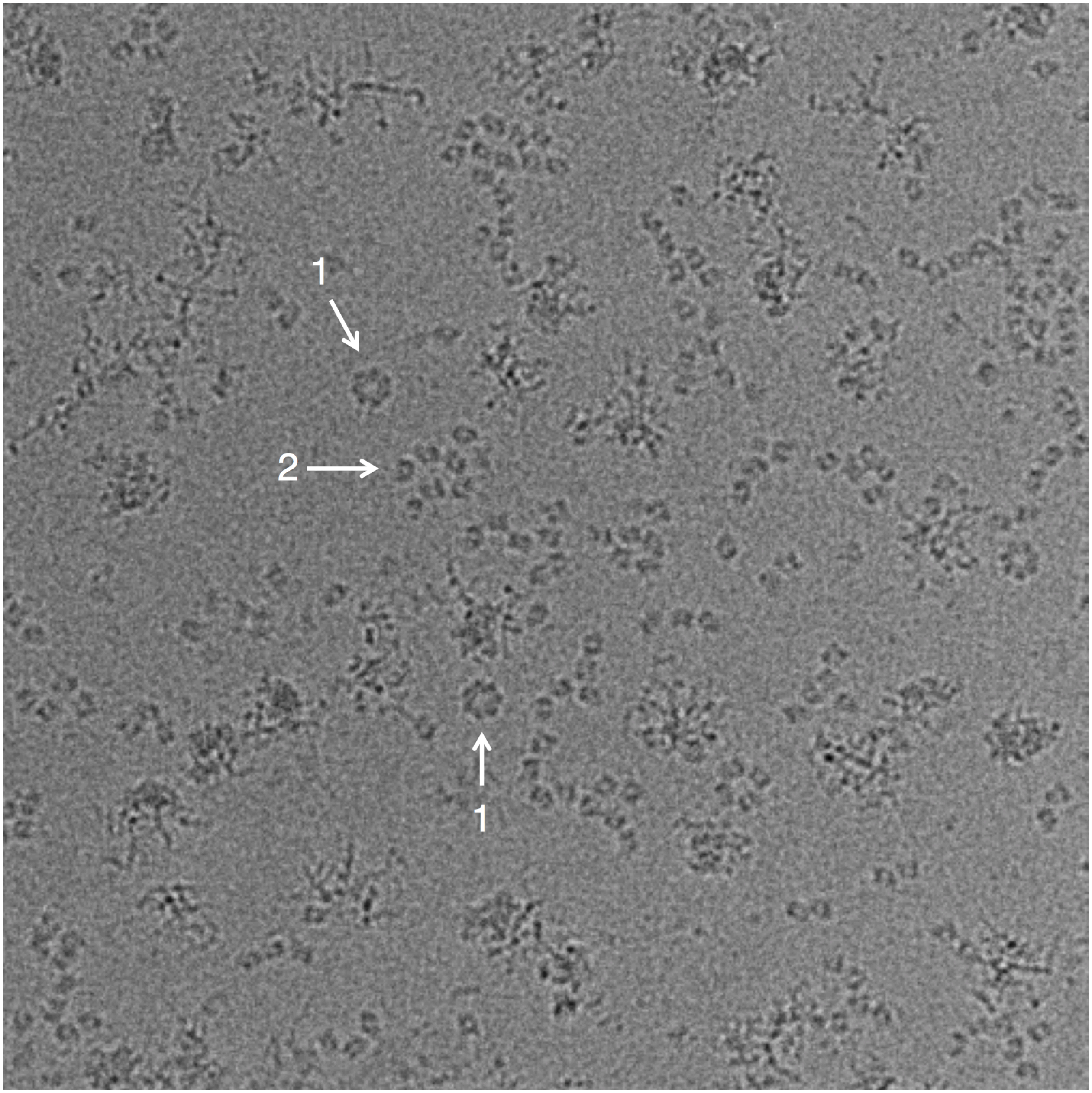

Figure S5. Oligomeric species. Electron micrograph showing two distinct species of *Op5*GH5, a hexameric or heptameric assembly (1) and the trimeric species (2).

Figure S6

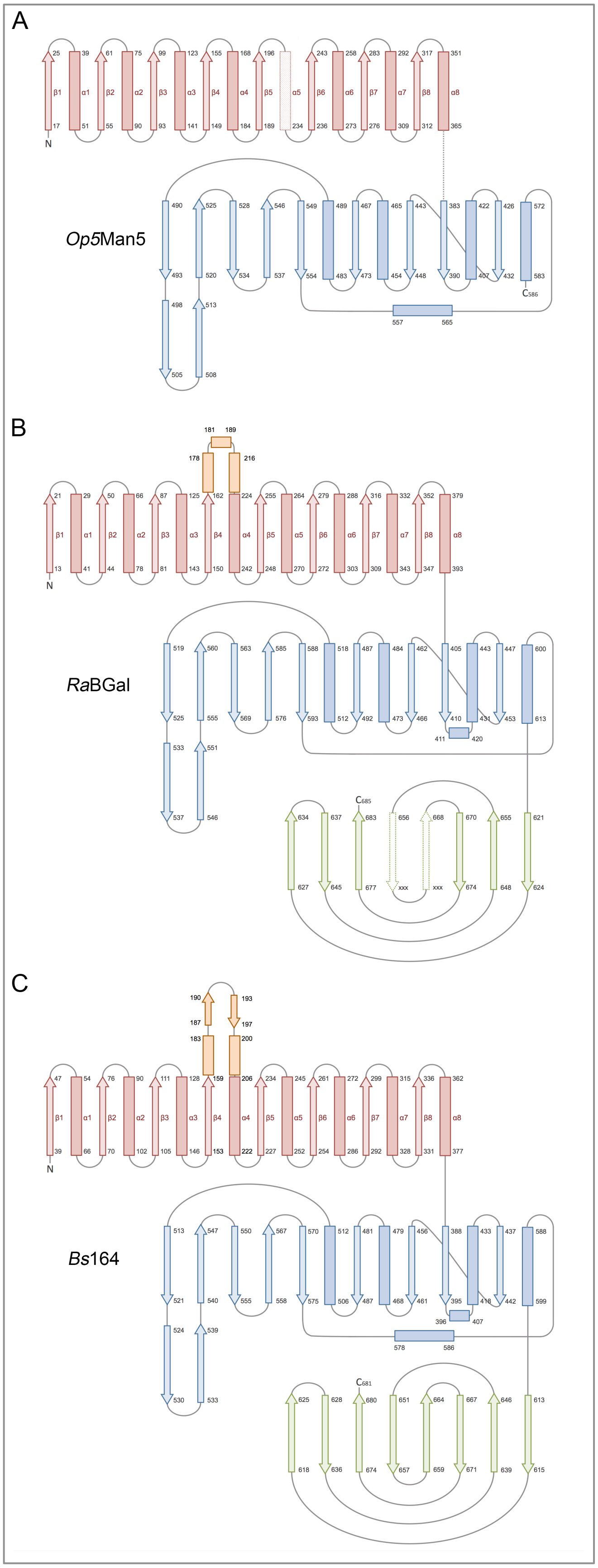

Figure S6. Topological similarity of *Op5*Man5, *Ra*BGal and *Bs*164. The N-terminal folds as a TIM barrel (domain A; red), and continues into a Rossmann-like mixed-sheet domain (domain B; blue). Except for a helical extension (orange) in the A-domain of *Ra*BGal and *Bs*164, and an additional peripheral α-helix in the B domain of *Op5*Man5, the topology of the A and B domains are identical in the three enzymes. The C-terminal domain (domain C; green) in *Ra*BGal and *Bs*164 features a Greek-key fold that is not present in *Op5*Man5. Two β-strands in the Greek-key domain of *Ra*BGal are partly disordered, which is indicated as dashed arrows. The unmodeled region in *Op5*Man5 comprising residues 210-221 is shown as a dashed, unfilled rectangle indicating a possible α-helix that is either very flexible or partly unfolded. A second unmodeled region is the linker peptide between domains A and B in *Op5*Man5 (residues 366-382). Note that the deposited *Bs*164 models have a sequence offset of +10 residues relative the sequence deposited in UniProt (entry I9SUA3), and the numbering used in the topology diagram is that of the models.

Figure S7

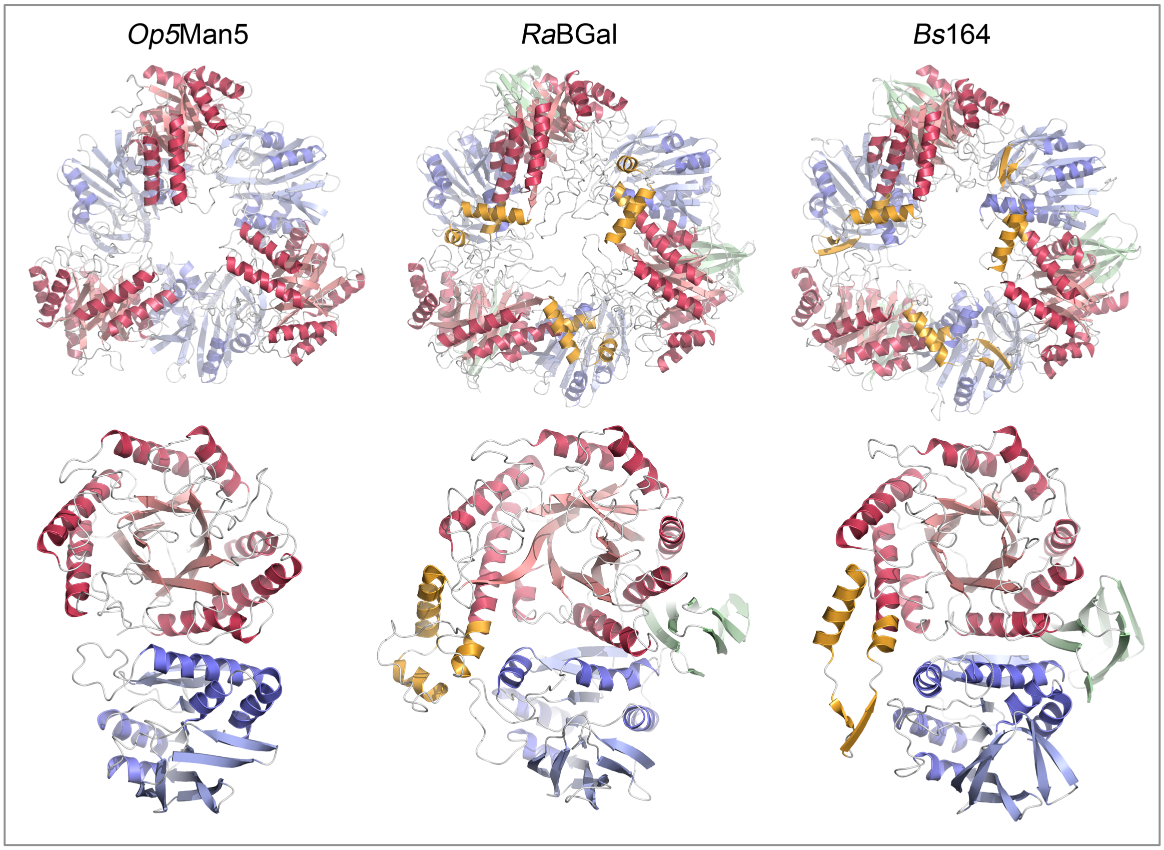

Figure S7. Structural comparison of *Op5*Man5, *Ra*BGal and *Bs*164. Overview of the trimer and monomer structure of *Op5*Man5 (this work), *Ra*BGal (PDB code 5E9A) and *Bs*164 (PDB code 6T75). The same colors are used as in Fig. S5.

Figure S8

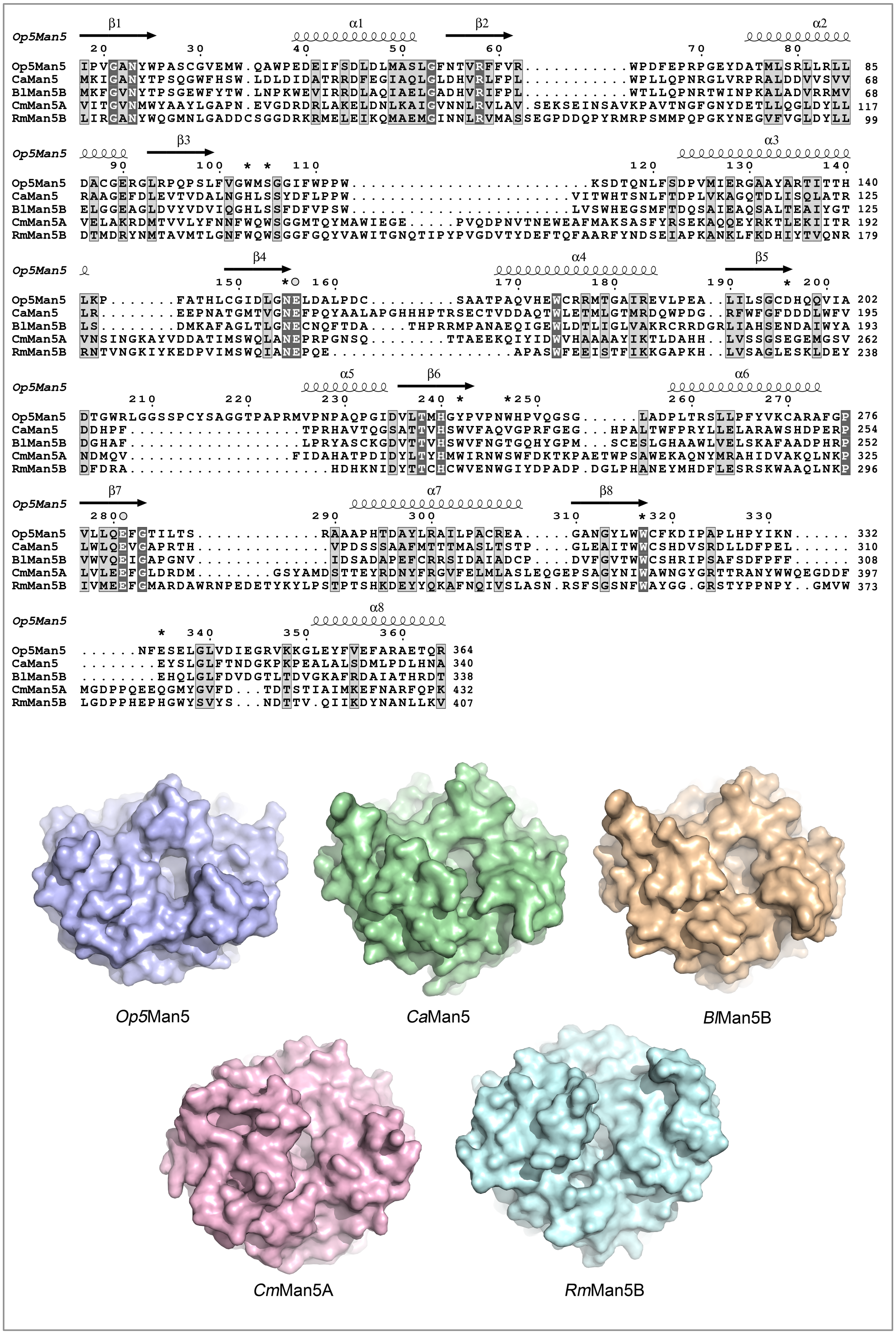

Op5Man5
IPVGANYWPASCGVEMW.QAWPEDEIFSDLDLMASLGFNTVRFFVR...........WPDFEPRPGEYDATMLSRLLRLL 85
CaMan5
MKIGANYTPSQGWFHSW.LDLDIDATRRDFEGIAQLGLDHVRLFPL...........WPLLQPNRGLVRPRALDDVVSVV 68
BlMan5B
MKFGVNYTPSGEWFYTW.LNPKWEVIRRDLAQIAELGADHVRIFPL...........WTLLQPNRTWINPKALADVRRMV 68
CmMan5A
VITGVNMWYAAYLGAPN.EVGDRDRLAKELDNLKAIGVNNLRVLAV.SEKSEINSAVKPAVTNGFGNYDETLLQGLDYLL 117
RmMan5B
LIRGANYWQGMNLGADDCSGGDRKRMELEIKQMAEMGINNLRVMASSEGPDDQPYRMRPSMMPQPGKYNEGVFVGLDYLL 99
DACGERGLRPQPSLFVGWMSGGIFWPPW.......................KSDTQNLFSDPVMIERGAAYARTITTH 140
RAAGEFDLEVTVDALNGHLSSYDFLPPW.......................VITWHTSNLFTDPLVKAGQTDLISQLATR 125
ELGGEAGLDVYVDVIQGHLSSFDFVPSW.......................LVSWHEGSMFTDQSAIEAQSALTEAIYGT 125
VELAKRDMTVVLYFNNFWQWSGGMTQYMAWIEGE.....PVQDPNVTNEWEAFMAKSASFYRSEKAQQEYRKTLEKIITR 192
DTMDRYNMTAVMTLGNFWQWSGGFGQYVAWITGNQTIPYPVGDVTYDEFTQFAARFYNDSEIAPKANKLFKDHIYTVQNR 179
LKP.......FATHLCGIDLGNELDALPDC.........SAATPAQVHEWCRRMTGAIREVLPEA..LILSGCDHQQVIA 202
LR........EEPNATGMTVGNEFPQYAALAPGHHHPTRSECTVDDAQTWLETMLGTMRDQWPDG..RFWFGFDDDLWFV 195
LS........DMKAFAGLTLGNECNQFTDA....THPRRMPANAEQIGEWLDTLIGLVAKRCRRDGRLIAHSENDAIWYA 193
VNSINGKAYVDDATIMSWQLANEPRPGNSQ........TTAEEKQIYIDWVHAAAAYIKTLDAHH..LVSSGSEGEMGSV 262
RNTVNGKIYKEDPVIMSWQIANEPQE..................APASWFEEISTFIKKGAPKH..LVSAGLESKLDEY 238
DTGWRLGGSSPCYSAGGTPAPRMVPNPAQPGIDVLTMHGYPVPNWHPVQGSG......LADPLTRSLLPFYVKCARAFGP 276
DDHPF.................TPRHAVTQGSATTVHSWVFAQVGPRFGEG...HPALTWFPRYLLELARAWSHDPERP 254
DGHAF.................LPRYASCKGDVTTVHSWVFNGTGQHYGPM...SCESLGHAAWLVELSKAFAADPHRP 252
NDMQV.................FIDAHATPDIDYLTYHMWIRNWSWFDKTKPAETWPSAWEKAQNYMRAHIDVAKQLNKP 325
DFDRA...................HDHKNIDYTTCHCWVENWGIYDPADP.DGLPHANEYMHDFLESRSKWAAQLNKP 296
VLLQEFGTILTS..............RAAAPHTDAYLRAILPACREA.....GANGYLWWCFKDIPAPLHPYIKN...... 332
LWLQEVGAPRTH..............VPDSSSAAFMTTTMASLTSTP....GLEAITWWCSHDVSRDLLDFPEL...... 310
VWVQEIGAPGNV..............IDSADAPEFCRRSIDAIADCP....DVFGVTWWCSHRIPSAFSDFPFF...... 308
LVLEEFGLDRDM........GSYAMDSTTEYRDNYFRGVFELMLASLEQGEPSAGYNIWAWNGYGRTTRANYWWQEGDDF 397
IVMEEFGMARDAWRNPEDETYKYLPSTPTSHKDEYYQKAFNQIVSLASN.RSFSGSNFWAYGG.GRSTYPPNPY..GMVW 373
......NFESELGLVDIEGRVKKGLEYFVEFARAETQR 364
........EYSLGLFTNDGKPKPEALALSDMLPDLHNA 340
........EHQLGLFDVDGTLTDVGKAFRDAIATHRDT 338
MGDPPQEEQGMYGVFD...TDTSTIAIMKEFNARFQPK 432
LGDPPHEPHGWYSVYS...NDTTV.QIIKDYNANLLKV 407
Op5Man5
CaMan5
BlMan5B
CmMan5A
RmMan5B

Figure S8. Comparison of the catalytic TIM-barrel domains of GH5 exo-β-1,4-mannosidases. (A) Structure-based sequence alignment of the catalytic TIM-barrel domains of *Op5*Man5, *Ca*Man5 (PDB 6GVB[31]), *Bl*Man5B (PDB 6MPA[47]), *Cm*Man5A (PDB 1UUQ[20]) and *Rm*Man5B (PDB 4LYP[17]). Surface representations of the TIM-barrel domains are shown to highlight the differences in shape of the entrance to the binding pockets.

Figure S9

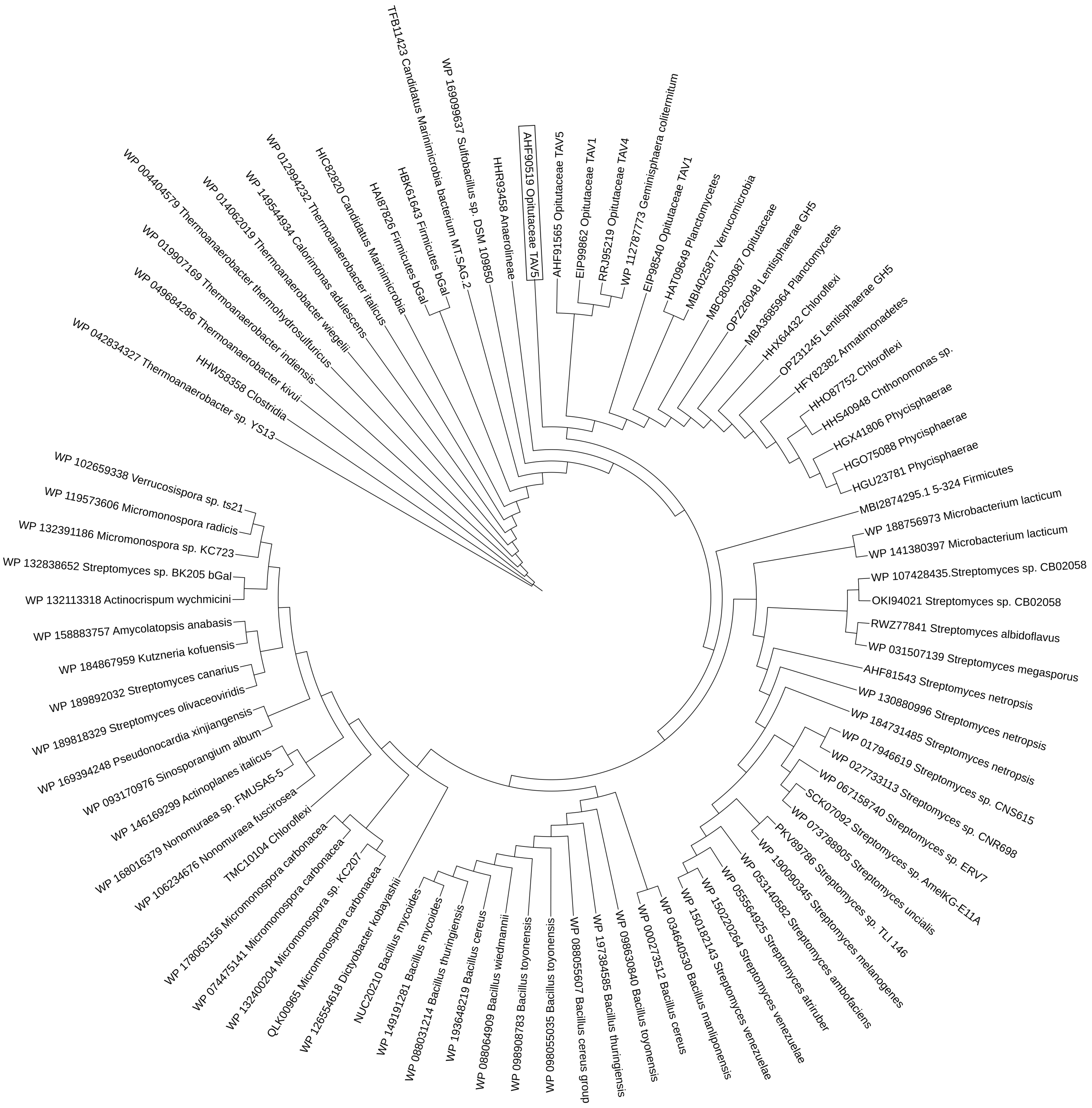

Figure S9. Phylogenetic tree. The *Op5*Man5 sequence (AHF90519, UniProt W0J1H8) is highlighted.

Figure S10

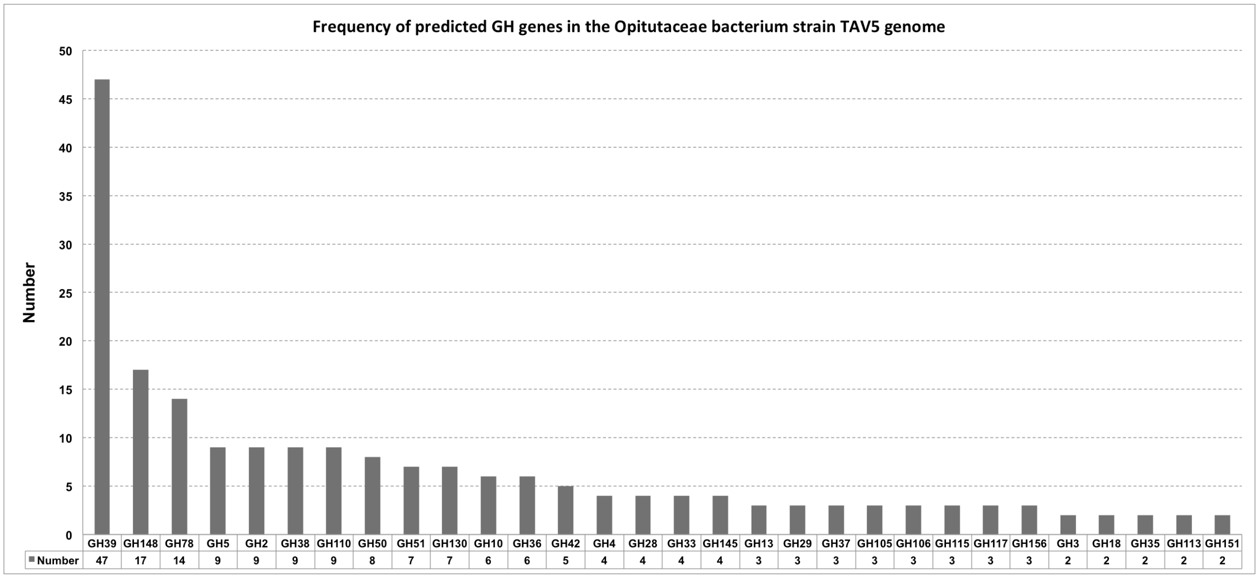

Figure S10. GH genes in the *Opitutaceae* bacterium strain TAV5 genome. The distribution of GH genes in the genome of *Opitutaceae* bacterium strain TAV5 according to GH family.

Figure S11

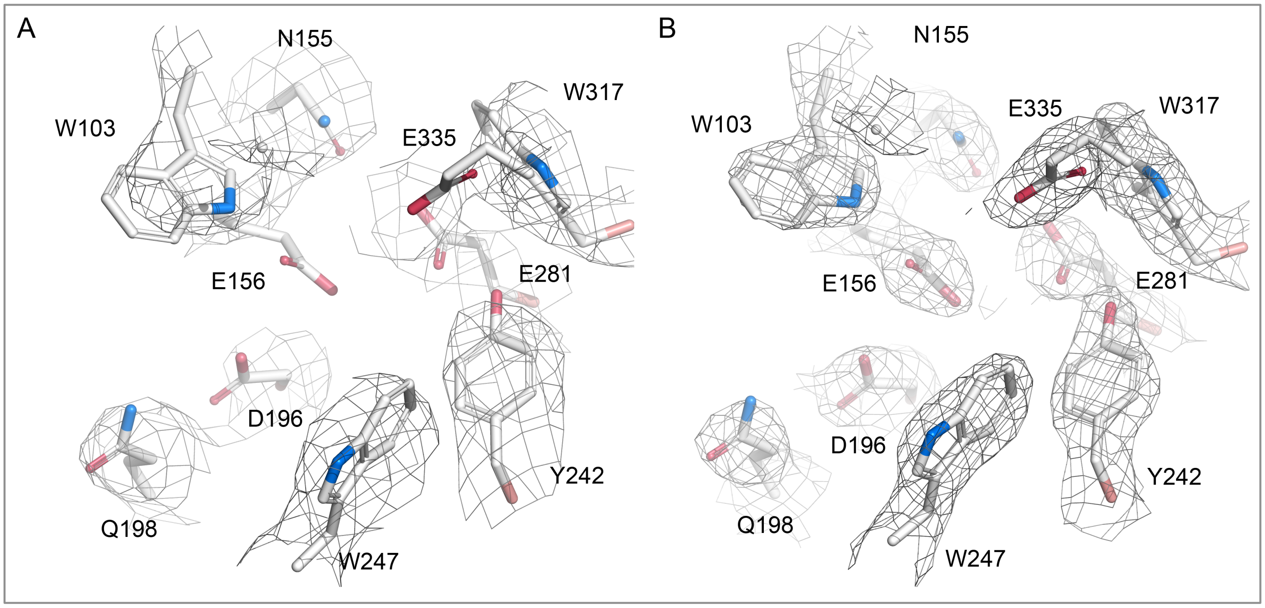

Figure S11. Quality of the cryo-EM and final X-ray map. Comparison of the map quality in the region of the active site. The 3.7 Å cryo-EM map (A) is of good overall quality, especially for aromatic residues, but lacks detectable signal for some side chains (E156, D196, E335), for which the 2.2 Å $2F_o$-F_c electron-density map (B) provided additional information.

Table S1. Reported activity on *p*NP-*β*Man for a selection of retaining glycoside hydrolases with exo-*β*-1,4-mannosidase activity (EC 3.2.1.25).

Enzyme name	Gene name	Organism name	UniProt accession	CAZy subfamily	[a]Molecular weight (g mol^{-1})	PDB accession	Oligomeric state	[b]K_m (mM)	[b]k_{cat} (s^{-1})	[b]k_{cat}/K_m (s^{-1} mM^{-1})	*Specific activity* U mg^{-1}	Transglycosylation activity	Reference	Digital object identifier
**GH5 (Bacteria)**														
*Op5*Man5	OPIT5_10225	*Opitutaceae* bacterium strain TAV5, Verrucomicrobia	W0J1H8	–	66,062	This work	homotrimer / hexamer	1.0	238.9	238.9	–	Yes	This work	
*Tth*Man5	Theth_0949	*Pseudothermotoga (Thermotoga) thermarum* strain DSM 5069, Thermotogae	F7YX66	19	70,687	[c]n.a.	oligomer	4.4	1924	441	–	n.a.	Shi *et al*. 2013	10.1186/1472-6750-13-83
*Ca*Man5_18	PAZ_c15370	*Cutibacterium acnes (Propionibacterium acnes),* Actinobacteria	A0A2B7I7A6	18	45,701	6GVB	homodimer	10.4	7.8	0.75	–	n.a.	Reichenbach et al. 2018	10.1371/journal.pone.0204703
*Bl*Man5B	BLD_0195 (BL1333)	*Bifidobacterium longum strain* DJO10A, Actinobacteria	B3DQP5	18	47,441	6MPA	homodimer	[d]n.a.	[d]n.a.	[d]n.a.	–	n.a.	Cordeiro et al 2019	10.1016/j.jmb.2018.12.017
*Cm*Man5A	man5A	*Cellvibrio mixtus strain* NCIMB 8633, Proteobacteria	Q6QT42	7	51,623	1UUQ	monomer	1.6	*0.033*	*0.021*	–	Yes	Dias et al 2004	10.1074/jbc.M401647200
**GH5 (Eukaryota, Fungi)**														
*Rm*Man5B	man5B	*Rhizomucor miehei* strain CAU432, zygomycete fungus	A0A075C6T6	7	51,321	4LYP, 4LYQ	monomer	n.a.	n.a.	n.a.	1.93	Yes	Zhou et al 2014	10.1107/S1399004714019762
*Lr*Man5B	LRAMOSA04528	*Lichtheimia ramosa,* zygomycete fungus	A0A077WZ93	–	50,996	n.a.	n.a.	1.38	19.7	14.3	–	n.a.	Xie et al. 2019	10.1016/j.jbiosc.2019.04.007
*Ac*Man5	acman5	*Lichtheimia (Absidia) corymbifera* strain D1, zygomycete fungus	A0A141PG32	–	49,797	n.a.	n.a.	n.a.	n.a.	n.a.	2.65	Yes	Li et a.. 2015	10.1016/j.molcatb.2015.09.018
**GH2 (Bacteria)**														
*Bt*Man2A	BT_0458	*Bacteroides thetaiotaomicron* strain VPI-548, Bacteroidetes	Q8AAK6	–	99,506	2JE8	homodimer	0.19	*128*	*667*	–	No	Tailford *et al*. 2007	10.1074/jbc.M610964200
*Tm*Man2	TM_1624	*Thermotoga maritima* strain MSB8, Thermotogae	Q9X1V9	–	92,373	n.a.	monomer	0.49	*8.4*	*17.2*	–	Yes	Zhang et al. 2009	10.1016/j.molcatb.2009.04.005
*Tn*Man2	man2	*Thermotoga neapolitana* strain DSM 5068, Thermotogae	Q93M25	–	92,197	n.a.	homodimer	3.1	*67.8*	*24.2*	–	n.a.	Duffaud et al. 1997	10.1128/AEM.63.1.169-177.1997
ManB	manB	*Thermobifida (Thermomonospora) fusca* strain TM5, Actinobacteria	Q8KLI9	–	94,228	n.a.	n.a.	0.18	*9.34*	*51.9*	–	Yes	Beki et al. 2003	10.1128/aem.69.4.1944-1952.2003
*Cf*Man2A	man2	*Cellulomonas fimi* strain ATCC 484, Actinobacteria	Q9XCV4	–	93,696	n.a.	monomer	0.3	[e]167	[d]500	–	n.a.	Stoll et al. 1999	10.1128/AEM.65.6.2598-2605.1999
Man2S27	bma	*Streptomyces sp. S27,* Actinobacteria	D2DFB5	–	92,580	n.a.	n.a.	0.23	305	1320	–	Yes	Shi et al. 2011	10.1016/j.enzmictec.2011.06.003
NixH	XCC2891	*Xanthomonas campestris pv. campestris* strain ATCC 3391, Proteobacteria	Q8P6S4	–	100,196	n.a.	n.a.	7.4	1552	209.8	–	n.a.	Dupoiron et al. 2015	10.1074/jbc.M114.624593
*Dt*Man	DICTH_1692	*Dictyoglomus thermophilum* strain DSM 3960, Firmicutes	B5YAN4	–	96,130	5N6U	homodimer	0.47	*298*	*617*	–	n.a.	Guillotin et al. 2017	10.1016/j.biochi.2017.03.020
–	–	*Paenibacillus polymyxa (Bacillus polymyxa)* strain A-8, Firmicutes	U5N3E2	–	96,836	n.a.	n.a.	n.a.	n.a.	n.a.	*38*	n.a.	Bai et al. 2014	10.1371/journal.pone.0111622
–	–	*Paenibacillus polymyxa (Bacillus)* strain A-8, Firmicutes, recombinant	U5N3E2	–	96,836	n.a.	n.a.	n.a.	n.a.	n.a.	*0.7*	n.a.	Bai et al. 2014	10.1371/journal.pone.0111622
**GH2 (Eukaryota)**														
MndA	mndA	*Aspergillus niger* strain ATCC 46890, ascomycete fungus	Q9UUZ3	–	104,390	n.a.	homodimer	0.30	*67.5*	*225*	–	Yes	Ademark et al. 1999	10.1016/s0168-1656(99)00172-8
MndA	mndA	*Aspergillus niger* strain ATCC 46890, ascomycete fungus	Q9UUZ3	–	104,390	n.a.	homodimer	0.46	*67.5*	*217*	–	Yes	Reddy et al. 2013	10.1016/j.febslet.2013.08.029
MndB	mndB	*Aspergillus (Emericella) nidulans* strain ATCC 38163, ascomycete fungus	Q5B7W2	–	96,367	n.a.	n.a.	0.22	*1.32*	*6.0*	–	n.a.	Reddy et al. 2013	10.1016/j.febslet.2013.08.029
bMann9	man9	*Thermothelomyces thermophilus* strain C1, ascomycete fungus	I2C092	–	97,352	n.a.	n.a.	0.41	15	37	–	n.a.	Dotsenko et al. 2012	10.1134/S0006297912110090
*Tr*Man2A	–	*Hypocrea jecorina (Trichoderma reesi)* strain Z12, ascomycete fungus	n.a.	–	n.a.	n.a.	homodimer, putative	0.12	[f]*5.2*	[e]*43*	–	n.a.	Kulminskaya et al. 1999	10.1016/S0141-0229(99)00056-3
*Th*Man2A	–	*Hypocrea lixii (Trichoderma harzianum)* strain IOC-3844, ascomycete fungus	A0A075B5H6	–	105,388	4CVU	monomer	n.a.	n.a.	n.a.	3.2	Yes	Nascimento et al. 2014	10.1111/febs.12894
ManbA	Manba	*Mus musculus* (lysosomal)	Q8K2I4	–	100,831	6DDU	monomer	1.42	72.2	*51*	–	n.a.	Gytz et al. 2019	10.1111/febs.14731
–	manba	*Capra hircus* (lysosomal), goat kidney	Q95327	–	101,386	n.a.	n.a.	9	n.a.	n.a.	1.08	n.a.	Frei et al. 1982	10.1042/bj2490871
–	manba	*Homo sapiens (lysosomal),* human serum	n.a.	–	(90,000)	n.a.	n.a.	2.7	n.a.	n.a.	n.a.	n.a.	Bernard et al. 1986	10.1016/0020-711x(86)90255-7
–	manba	*Homo sapiens (lysosomal),* human urine	n.a.	–	(100,000)	n.a.	n.a.	2.4	n.a.	n.a.	n.a.	n.a.	Bernard et al. 1986	10.1016/0020-711x(86)90255-7
–	manba	*Homo sapiens* (lysosomal), human placenta	O00462	–	(75,000)	n.a.	n.a.	2.2	n.a.	n.a.	n.a.	n.a.	Noeske & Mersmann 1983	10.1515/bchm2.1983.364.2.1645
–	manba	*Homo sapiens* (lysosomal), human placenta	O00462	–	100,895	n.a.	n.a.	2.5	n.a.	n.a.	12.7	n.a.	Samra & Athar 2008	Acta Biochim. Pol. 55, 479-490, 2008
*Ak*Mnsd	AkMnsd	*Aplysia kurodai* (gut), Kuroda's sea hare	H1AFK3	–	105,059	n.a.	n.d.	0.10	*6.2*	*62*	–	n.a.	Zahura et al 2012	10.1016/j.cbpb.2012.02.003

Table S1. Cont.

Enzyme name	Gene name	Organism name	UniProt accession	CAZy subfamily	[a]Molecular weight (g mol^{-1})	PDB accession	Oligomeric state	[b]K_m (mM)	[b]k_{cat} (s^{-1})	[b]k_{cat}/K_m (s^{-1} mM^{-1})	*Specific activity* U mg^{-1}	Transglycosylation activity	Reference	Digital object identifier
**GH1 (Archaea)**														
BmnA	bmnA	*Pyrococcus furiosus* strain DSM 3638	Q51733	–	59,069	n.a.	tetramer	0.79	*31*	*39*	–	n.a.	Bauer et al 1996	10.1074/jbc.271.39.23749
PFTG	PF0356	*Pyrococcus furiosus* strain DSM 3638	Q8U3U9	–	56,327	n.a.	tetramer	0.09	0.30	3.0	–	Yes	Park et al. 2011	10.1016/j.nbt.2011.05.002
BglB	PH0501	*Pyrococcus horikoshii* strain OT3	O58237	–	56,458	n.a.	tetramer	0.44	4.2	9.8	–	n.a.	Kaper et al. 2002	10.1021/bi011935a
TkβGly	–	*Thermococcus kodakarensis* strain KOD1	Q9YGB8	–	56,266	n.a.	homodimer	1.1	51	47	–	n.a.	Hwa et al. 2015	10.1016/j.enzmictec.2015.05.002
**GH1 (Eukaryota, Plantae)**														
HvBII	–	*Hordeum vulgare subsp. vulgare,* barley	B5A496	–	57,425	n.a.	n.a.	0.25	3.1	12.7	–	Yes	Kuntothom et al. 2010	10.1021/bi101112c
Os7BGlu26	OsI_27163	*Hordeum vulgare subsp. indica,* barley	A2YPH1	–	58,498	4JHO	monomer	0.48	0.35	0.71	–	n.a.	Tankrathok et al. 2013	10.1107/S0907444913020568
LeMside	–	*Solanum lycopersicum (Lycopersicon esculentum),* tomato	Q8VWL8	–	59,288	n.a.	n.a.	0.55	*10*	*18*	–	n.a.	Mo & Bewley 2002	10.1007/s00425-001-0725-x
BGLU44	BGLU44	*Arabidopsis thaliana,* thale cress	Q9LV33	–	58,984	n.a.	n.a.	0.43	*6*	*14*	–	n.a.	Xu et al. 2004	10.1007/s11103-004-0790-1
**GH113 (Bacteria)**														
*Ri*GH113	ROSINTL182_05483	*Roseburia intestinalis* strain L1-82		–	36,057	n.a.	n.a.	n.a.	n.a.	n.a.	n.a.	n.a.	La Rosa et al. 2019	10.1038/s41467-019-08812-y
**GH164 (Bacteria)**														
*Bs*164	HMPREF1071_03408	*Bacteroides salyersiae* strain CL02T12C01	I9SUA3	–	76,732	6T5O, 6T75	homotrimer	3.6	40	11	–	n.a.	Armstrong & Davies 2020	10.1074/jbc.RA119.011591
**Not classified in CAZy**														
–	–	*Bacillus mannanilyticus* strain DSM 16130 (AM-001), bacteria, firmicutes	n.a.	–	(94,000)	n.a.	n.a.	1.3	n.a.	n.a.	38.8	Yes	Akino et al. 1988	10.1080/00021369.1988.10868864
–	–	*Tremella fuciformis,* basidiomycete fungus	n.a.	–	(140,000)	n.a.	n.a.	2.1	n.a.	n.a.	0.21	n.a.	Sone & Misaki 1978	10.1093/OXFORDJOURNALS.JBCHEM.A132003
–	–	*Laetiporus (Polyporus) sulphureus,* basidiomycete fungus	n.a.	–	(64,000)	n.a.	n.a.	1.6	n.a.	n.a.	4.2	n.a.	Wan et al. 1976	10.1016/s0021-9258(17)33308-2
–	–	*Athelia (Sclerotium) rolfsii,* basidiomycete fungus	–	–	(57,500)	n.a.	n.a.	n.a.	n.a.	n.a.	*41*	n.a.	Gübitz et al. 1996	10.1016/S0960-8524(96)00093-4
GM-1 (isoenzyme)	–	*Phlebia radiata* strain ATCC 64658, basidiomycete fungus	n.a.	–	(104,600)	n.a.	n.a.	0.3	*74*	*247*	–	n.a.	Prendecka et al. 2007	Pol. J. Microbiol. 56, 139-147, 2007
GM-2 (isoenzyme)	–	*Phlebia radiata* strain ATCC 64658, basidiomycete fungus	n.a.	–	(89,500)	n.a.	n.a.	1.8	*72*	*40*	–	n.a.	Prendecka et al. 2007	Pol. J. Microbiol. 56, 139-147, 2007
–	–	*Thermoascus aurantiacus* Miehe, ascomycete fungus	n.a.	–	(99,900)	n.a.	monomer	1.1	6.1	*5.5*	–	Yes	Gomes et al. 2007	10.1016/j.enzmictec.2006.08.011
–	–	*Helix pomatia,* Roman snail	n.a.	–	(94,000)	n.a.	n.a.	1.43	*159*	*111*	–	n.a.	McCleary 1983	10.1016/0008-6215(83)88314-1
–	–	*Helix aspersa* Müller *(Cornu aspersum),* brown garden snail	n.a.	–	(77,800)	n.a.	monomer	0.77	*64*	*83*	–	n.a.	Charrier & Rouland 2001	10.1002/jez.1042
–	–	*Achatina fulica,* giant African snail	n.a.	–	n.a.	n.a.	n.a.	6.5	n.a.	n.a.	6.67	n.a.	Sugahara et al. 1972	10.1016/0005-2744(72)90344-0
–	–	*Turbo cornutus,* horned turban sea snail	n.a.	–	n.a.	n.a.	n.a.	7.1	n.a.	n.a.	3.8	n.a.	Muramatsu & Egami 1967	10.1093/oxfordjournals.jbchem.a128726
–	–	*Aplysia fasciata,* mottled sea hare	n.a.	–	(130,000)	n.a.	homodimer	2.4	*192*	*80*	–	Yes	Andreotti et al. 2005	10.1016/j.jbiotec.2005.06.003
–	–	*Hordeum vulgare,* malted barley	n.a.	–	(88,000)	n.a.	n.a.	0.3	n.a.	n.a.	0.6	n.a.	Houston et al. 1974	10.1016/0005-2744(74)90052-7
–	–	*Lactuca sativa L. cv. Grand Rapids,* garden lettuce	n.a.	–	n.a.	n.a.	n.a.	0.3	n.a.	n.a.	n.a.	n.a.	Ouellette & Bewley 1986	10.1007/BF00392128
–	–	*Cyamopsis tetragonolobus* (guar seeds)	–	–	(59,000)	n.a.	n.a.	0.5	*15*	*30*	15.6	n.a.	McCleary 1982	Meth. Enzymol. 160, 589-595, 1982
–	–	*Gallus gallus,* hen oviduct	n.a.	–	(100,000)	n.a.	n.a.	2.9	n.a.	n.a.	n.a.	n.a.	Sukeno et al. 1972	10.1021/bi00758a026

[a] Theoretical molecular weight of a protein monomer derived from the amino-acid sequence. Numbers in soft brackets refer to the experimentally determined molecular weight by SDS-PAGE and is given when the sequence is not available.

[b] Italized values were derived estimates calculated from the data reported in the original reference

[c] n.a., information not available

[d] Activity confirmed but substrate saturation not reached, which prevented deduction of the catalytic constants

[e] Substrate inhibition was observed and the values are estimates.

[f] The derived values assume that the k_{cat} value is 2.95 10^{-3} μmol $μg^{-1}$ min^{-1}, and not 2.95 10^{-3} μmol min $μg^{-1}$ as given in the original reference

Table S2. Data collection, phasing and refinement statistics

**Data collection**[a]	
Synchrotron, beamline	Diamond Light Source, *I*03
Wavelength (Å)	0.826561
Space group / mol per a.s.u.	$P2_12_12_1$ / 3 (one homotrimer)
Cell dimensions: *a*, *b*, *c* (Å), α, β, γ (°)	102.14, 162.45, 168.18, 90, 90, 90
Resolution (Å), nominal	49.15–2.20 (2.30–2.20)
Unique reflections	141664 (17459)
R_{sym}	0.107 (1.865)
$I / \sigma I$	11.9 (1.3)
Completeness (%)	99.7 (99.4)
Redundancy	13.3 (12.6)
CC(1/2)[b]	99.9 (76.8)
Wilson *B* factor (Å^2)	45.16
**Refinement**	
Resolution (Å)	48.87–2.20 (2.28–2.20)
Completeness	99.64 (99.39)
No. reflections work	141605 (13961)
No. reflections free	2012 (197)
R_{work}	0.203 (0.385)
R_{free}	0.227 (0.401)
N^o. non-hydrogen atoms: all, protein, water	13299, 13134, 165
N^o. protein residues, all chains	1662
Missing residues chain A, B, C	41, 38, 41
Mean *B* factors protein, water (Å^2)	56.9, 52.0
R.m.s deviation bond lengths (Å), bond angles (°)	0.008, 0.89
Ramachandran, MolProbity: allowed, favored, outliers (%)	99.94, 97.14, 0.06
PDB ID	7BOB

[a] Statistics for outer-shell reflections are given in parentheses.

[b] Percentage of correlation between intensities from random half-datasets as given by XSCALE. Values given represent correlations significant at the 0.1% level.[48]
